# Energetic Co-Crystal of a Primary Metal-Free Explosive with BTF. Ideal Pair for Co-Crystallization

**DOI:** 10.3390/molecules26247452

**Published:** 2021-12-09

**Authors:** Kyrill Yu. Suponitsky, Ivan V. Fedyanin, Valentina A. Karnoukhova, Vladimir A. Zalomlenkov, Alexander A. Gidaspov, Vladimir V. Bakharev, Aleksei B. Sheremetev

**Affiliations:** 1 A.N. Nesmeyanov Institute of Organoelement Compounds, Russian Academy of Sciences, 28 Vavilov Street, 119991 Moscow, Russia; octy@xrlab.ineos.ac.ru (I.V.F.); v_a_k_h_h@mail.ru (V.A.K.); 2 Chemistry Department, Samara State Technical University, 443100 Samara, Russia; vaz.sgtu@gmail.com (V.A.Z.); knil@sstu.smr.ru (A.A.G.); knilsstu@gmail.com (V.V.B.); 3 N.D. Zelinsky Institute of Organic Chemistry, Russian Academy of Sciences, 119991 Moscow, Russia; sab@ioc.ac.ru

**Keywords:** high energetic materials, co-crystallization, X-ray diffraction, crystal packing analysis, periodic calculation, electron density

## Abstract

Co-crystallization is an elegant technique to tune the physical properties of crystalline solids. In the field of energetic materials, co-crystallization is currently playing an important role in the engineering of crystals with improved performance. Here, based on an analysis of the structural features of the green primary explosive, tetramethylammonium salt of 7-oxo-5-(trinitromethyl)-4,5,6,7-tetrahydrotetrazolo[1,5-a][1,3,5]triazin-5-ide (**1**), a co-former such as the powerful secondary explosive, benzotrifuroxan (BTF, **2**), has been proposed to improve it. Compared to the original **1**, its co-crystal with BTF has a higher detonation pressure and velocity, as well as an initiating ability, while the impact sensitivity and thermal stability remained at about the same level. Both co-formers, **1** and **2**, and co-crystal **3** were characterized by single-crystal X-ray diffraction and their crystal packing was analyzed in detail by the set of approaches, including periodic calculations. In the co-crystal **3**, all intermolecular interactions were significantly redistributed. However, no new types of intermolecular interactions were formed during co-crystallization. Moreover, the interaction energies of structural units in crystals before and after co-crystallization were approximately the same. A similar trend was observed for the volumes occupied by structural units and their densifications. The similar nature of the organization of the crystals of the co-formers and the co-crystal gives grounds to assert that the selected co-formers are an ideal pair for co-crystallization, and the invariability of the organization of the crystals was probably responsible for the preservation of some of their properties.

## 1. Introduction

A key aspiration of crystal engineering is to create functional materials with task-specific physical and/or chemical properties. A promising approach to achieve this is two-component co-crystallization producing bimolecular complexes [1,2,3,4,5,6,7,8]. However, in order to be able to purposefully influence the specific properties of co-crystals, it is important to understand at least the main factors that determine the supramolecular assembly of the principal molecule with a co-former through the intermolecular interactions. This is not an easy task. Although the co-crystallization technology, providing access to various molecular complexes, is under evolution, most studies focus on modifications of the pharmaceuticals [9,10,11,12,13]. The increased interest of the pharmaceutical industry in co-crystals is due to the expansion of opportunities for improving the physicochemical properties of known active pharmaceutical ingredients (API) without compromising the pharmaceutical activity. Typically, in pharmaceutical co-crystals, one component is an API, while the other one does not exhibit pronounced biological activity). On the other hand, drug–drug co-crystals are rare complexes wherein the co-former is not inert but is an API with independent bioactivity [10,11,12,13].

In the case of the design of new energetic materials by means of co-crystallization, both the use of inert co-former and energetic one is widely exploited. An inert co-former or a co-former, whose performance is significantly worse than that of the parent component can be utilized to improve those properties which are responsible for the safety of energetic materials [14,15,16,17,18,19,20]. However, it should be noted that the inert co-formers are not always suitable for use. Such co-formers can lead to a degradation of the main effects that are important for particular applications. In fact, selecting a useful co-former for an energetic compound is a challenge. Co-crystallization should lead to an improvement in some of the desired properties without significant loss of the other important properties.

The performance of an explosive is appreciated by its detonation velocity (D) and detonation pressure (P_CJ_) [21,22], and for a propellant by its specific impulse (I_SP_) [23,24]. These parameters are determined by the oxygen coefficient (α), enthalpy of formation (ΔH_f_), and density (ρ). The higher is the oxygen coefficient, enthalpy of formation, and density, the better would be the performance [21,22,23,24]. A candidate must demonstrate a suitable sensitivity toward heat, shock, friction, and electrostatic discharge, resistance to light, as well as stability during storage. It must also be compatible with other components of energetic materials, have an acceptable morphology, and be suitable for pressing, etc. From a practical standpoint, the synthetic route to a new energetic compound should be concise, reliable, safe, and scalable, and precursors should be inexpensive. Following these guidelines, many efforts have focused on the investigation of co-crystallization in which both co-formers are energetic compounds (see, for instance, refs. [25,26,27,28,29,30,31,32]. In particular, co-crystallization of the diacetone diperoxide (DADP) with 1,3,5-triiodo-2,4,6-trinitrobenzene (TITNB) led to a dramatic improvement of sensitivity which appeared to be lower than that in both co-formers [33].

In an earlier communication we disclosed our initial results on the discovery of energetic 5-(trinitromethyl)tetrazolo[1,5-a][1,3,5]triazine structures [34], among which tetramethylammonium salt of 7-oxo-5-(trinitromethyl)-4,5,6,7-tetrahydrotetrazolo[1,5-a][1,3,5]triazin-5-ide (**1**) is a potential metal-free primary explosive. However, despite the acceptable sensitivity to heat and shock, the density and energetic performance (see below) of this compound should be increased. Note that the design and synthesis of new green energetic compounds with high density and improved energetic properties has been the focus of recent research in laboratories around the world [35,36]. Due to its inherent properties and elemental composition, compound **1** has been identified as an attractive co-former for the development of improved green energetic materials.

**Scheme 1:**
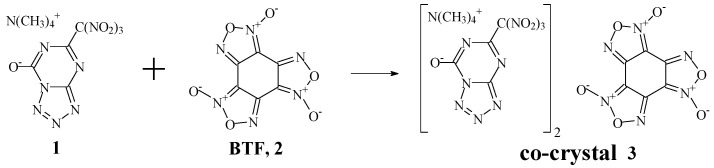
Schematic view of co-crystal obtaining.

Herein, salt **1** was investigated by the single-crystal X-ray diffraction. The crystal packing analysis of salt **1** made it possible to choose a suitable co-former, namely, benzotrifuroxan (BTF, **2**), to obtain a new energetic co-crystal **3** (Scheme 1). The structural peculiarities and properties of the original co-formers and the co-crystal are compared.

## 2. Experimental and Computational Part

**Caution!** Although we have encountered no difficulties during the preparation and handling of these compounds, they are potentially explosive energetic materials. Manipulations must be carried out by using appropriate standard safety precautions.

### 2.1. Preparation of the Co-Crystal ***3***

The starting co-formers, tetramethylammonium 5-(trinitromethyl)tetrazolo[1,5-a][1,3,5]triazin-7-olate (**1**) [34] and benzotrifuroxan (BTF, **2**) [37], were obtained according to published procedures. BTF (1 eq) and salt **1** (2 eq) were added to ethanol (50 eq) and heated with stirring until complete dissolution. The solution was concentrated by rotary evaporation before crystals began to form. The remaining solvent was allowed to evaporate at room temperature. The co-crystal **3**, consisting of two molecules of salt **1** and one molecule of BTF was obtained as a fine crystalline residue. IR (KBr): 1709, 1652, 1616, 1589, 1569, 1519, 1483, 1417, 1315, 1294, 1159, 1112, 1078, 960, 946, 925, 846, 804, 786 cm^−1^. Anal. calcd. for C_22_H_24_N_26_O_20_ (972.60): C 27.17, H 2.49, N 37.44; found C 27.28, H 2.53, N 37.35.

### 2.2. Thermal Analysis

Differential scanning calorimetry (DSC) was performed with a DSC 500 instrument [38]. The sample (1–2 mg) was heated from room temperature to 350 °C at a heating rate of 8 Kmin^−1^ in a nitrogen atmosphere (30 mL min^−1^).

### 2.3. Sensitivity Test

The sensitivity to impact of all materials was tested using a drop-hammer style instrument K-44-1M designed for handling primary explosives [21,39]. The impact sensitivity is given here as the impact energy *E*_im_, at a height of h_100%_ (maximum drop height is 50 cm), at which the impact with a freefalling 0.307 kg drop weight will provoke detonation with a 100% probability. For testing, a 20 mg sample was placed in a steel cap of the primer-igniter (inner diameter 5.58 ± 0.008 mm, outer diameter 6.10 ± 0.005 mm, height 2.45 ± 0.005 mm) and pre-pressed at a pressure of 100 MPa. The equipped cap was installed in a stand at the base of the pile driver, a steel ball (diameter 5.55 ± 0.007 mm) was placed on the energetic sample, which was struck when the load was dropped from a given height. A reproducible h_100%_ was obtained by striking the samples in 25 trials per energetic material.

### 2.4. Single Crystal X-ray Diffraction

Crystals of co-formers, **1** and **2**, and co-crystal **3,** suitable for single-crystal X-ray diffraction, were obtained by dissolving the compounds in a minimum amount of ethanol held at room temperature, followed by filtration of the crystals after the volume of the solvent had been reduced. In spite of earlier studies on structural peculiarities of BTF [40], we carried out its X-ray diffraction study in order to have all three experimental datasets made at the same conditions and on the same diffractometer.

X-ray experiments for compounds **1**, **2**, and **3** were carried out using a SMART APEX2 CCD diffractometer (λ(Mo-Kα) = 0.71073 Å, graphite monochromator, *ω*-scans) at 100 K. Collected data were processed by the SAINT and SADABS programs incorporated into the APEX2 program package [41]. The structures were solved by the direct methods and refined by the full-matrix least-squares procedure against *F*^2^ in anisotropic approximation. The refinement was carried out with the SHELXL program [42]. The CCDC numbers (2121440, 2121441, 2121442 for **1**, **2**, **3**, respectively) contain the supplementary crystallographic data for this paper. These data can be obtained free of charge via www.ccdc.cam.ac.uk/data_request/cif (accessed on 28 October 2021). Single crystals of salt **1** were obtained in the form of pale yellow thin plates of low quality due to significant disorder (details on the refinement are given in the Supplementary Materials). In the following discussion, we use those data only to define types of intermolecular interactions and to describe anion conformation.

Crystallographic data for **1**: C_4_N_9_O_7_^−^·C_4_H_12_N^+^ are monoclinic, space group *P*2_1_/*n*: *a* = 6.267(2) Å, *b* = 17.911(6) Å, *c* = 26.751(9) (5) Å, *β* = 93.399(7)°, *V* = 2997.4(18) Å^3^, *Z* = 8, *M* = 360.28, *d*_cryst_ = 1.597 g⋅cm^−3^. *wR*2 = 0.2378 calculated on *F*^2^*_hkl_* for all 5291 independent reflections with 2*θ* < 50.0°, (*GOF* = 1.003, *R* = 0.0822 calculated on *F_hkl_* for 1614 reflections with *I* > 2*σ*(I)).

Crystallographic data for **2**: C_6_N_6_O_6_ are orthorhombic, space group *Pna*2_1_: *a* = 6.8759(3) Å, *b* = 19.1923(8) Å, *c* = 6.5101(3) Å, *V* = 859.10(7) Å^3^, *Z* = 4, *M* = 252.12, *d*_cryst_ = 1.949 g⋅cm^−3^. *wR*2= 0.0699 calculated on *F*^2^*_hkl_* for all 2512 independent reflections with 2*θ* < 60.1°, (*GOF* = 1.045, *R* = 0.0265 calculated on *F_hkl_* for 2421 reflections with *I* > 2*σ*(I)).

Crystallographic data for **3**: 2C_4_N_9_O_7_^−^·2C_4_H_12_N^+^·C_6_N_6_O_6_ are triclinic, space group *P*-1: *a* = 6.8749(3) Å, *b* = 14.8319(6) Å, *c* = 20.4613(8) Å, α= 70.4870(10)° *βββ* = 83.7430(10)°, γ= 87.6940(10)° *V* = 1954.83(14) Å^3^, *Z* = 2, *M* = 972.67, *d*_cryst_ = 1.652 g⋅cm^−3^. *wR*2= 0.1221 calculated on *F*^2^*_hkl_* for all 9405 independent reflections with 2*θ* < 56.0°, (*GOF* = 1.020, *R* = 0.0467 calculated on *F_hkl_* for 6725 reflections with *I* > 2*σ*(I)).

### 2.5. Methodology of Crystal Structure Investigation

Two approaches were used to analyze the crystal packing. The first is a combination of geometrical and energetic approaches. Based on visual inspection of the close and shortened intermolecular contacts, one can define the type of interaction of the central molecule in a crystal with its closest environment. Such a qualitative analysis can be supplemented by an estimation of the energy between the central molecule and each molecule from its closest environment (pair interaction energy). The latter can be done in terms of atom–atom potentials, quantum chemical calculations of dimers (molecular pairs) at different levels of theory, semi-empirical methods based on electron density distribution, such as PIXEL or CE-B3LYP, “Atoms in molecules” (AIM) topological theory [43,44,45,46,47,48,49]. All those methods can be successfully used for crystals built up of neutral molecules. For the crystal structures of this study incorporating ionic structural units, the standard set of atom-atom potential parameters might be insufficient. Estimation of the unit…unit interaction energy with an ab initio method by calculation of dimeric associates with subsequent use of the *E*_int_ = *E*_AB_ − *E*_A_ − *E*_B_ formula, or use of the semi-empirical methods such as CE-B3LYP results in correct interaction energies. However, these values are not particularly useful in comparison of different associates in different structures or in the calculation of the lattice energies due to the long-range character of electrostatic forces [50]. With this respect, the use of AIM theory seems to be the method of choice as it is the only method that estimates the energy of a particular bonding interaction irrespective of the nature of the interacting units. An additional advantage is that the method can be based on both experimental and theoretical electron density distribution. Unfortunately, the quality of single crystals of salt **1** and its co-crystal with BTF (**3**) turned out to be insufficient for multipole refinement and reliable estimation of electron density distribution function.

Periodic ab initio calculations of the crystal structures of the co-crystal **3**, the parent salt **1** and the pure BTF **2** were carried out with the CRYSTAL17 program [51]. The dispersion-corrected PBE0-D3 functional [52,53] was used in combination with POB-TZVP basis set [54]. For all calculations, a shrinking factor 2 2 2 for the Monkhorst-Pack grid yielded in 8 k-points in the irreducible Brillouin zone. Atomic positions were optimized using the experimental unit cell parameters and symmetry. To model two distinct positions of the disordered trinitromethyl fragment in the pure salt structure, two separate geometries corresponding to different components of the disorder were optimized, which indeed converged to different molecular conformations. An energy difference of 2.1 kcal/mol between the structures indicates that the conformation corresponding to the major disordered component is more favorable. An agreement between theoretical and experimental structure is excellent for BTF, is good for the co-crystal, and is satisfactory for the salt (see Supplementary Materials for details).

For calculation of the lattice and cohesion energies for the pure BTF, the energy of the isolated BTF molecule was calculated for the crystal geometry and after optimization. In addition, the calculation of the isolated molecule in the nearly-the-crystal basis set was performed (MOLEBSSE keyword) to account for the basis set superposition error via the counterpoise approach [55]. The AIM topological analysis of the electron density distribution ρ(r) and the integration of atomic properties were carried out with the TOPOND program [56] incorporated into the CRYSTAL17 code. To compare the energy of individual bonding interatomic interactions, we used an empirical Espinosa–Molins–Lecomte (EML) correlation: E_EML_ = −0.5a_0_^3^*v*(**r**), where E_EML_ is the interaction energy, a_0_ is Bohr radius and *v*(**r**) is potential energy density at the bond critical point (BCP) [57]. The theoretical justification for this correlation and its possible limitations was proposed [58].

Hirshfeld surface analysis was performed using the CrystalExplorer package [59] utilizing the underlying TONTO program [60].

The second approach for crystal packing study relies on recently proposed densification analysis based on Δ_OED_ (overlap of electron density) criterion [61,62,63]. It is assumed that upon crystal formation, molecules interact with each other by means of overlap of their electron densities. It means that the volume of the isolated molecule is larger than that of the molecule in a crystal. Similarly, the density of an isolated molecule (*d*_mol_) is lower than that in a crystal (*d*_cryst_). The latter is the density of the crystal structure obtained from the X-ray experiment. In other words, upon crystal structure formation, the molecule is densified. Therefore, the Δ_OED_ criterion defined as

Δ_OED_ = *d*_cryst_ − *d*_mol_

would characterize a degree of molecular densification and, therefore, tightness of a crystal packing. It should be noted that the Δ_OED_—based approach can be applied not only to crystals that contain one molecule in an asymmetric unit cell, but also to any crystals, salts, solvates, co-crystals and their structural units as well as to any molecular fragments or functional groups [64,65]. More details on the estimation of Δ_OED_ are given in the Supplementary Materials.

In the following discussion, we use energies estimated by the EML correlation and molecular volumes calculated by the AIM approach based on theoretically obtained electron density. For BTF crystal which does not contain charged structural units, intermolecular energies are also estimated using the *E*_int_ = *E*_AB_ − *E*_A_ − *E*_B_ formula. Here *E*_AB_ is the energy of the dimer built up of the central BTF molecule and one of the neighboring molecules in the crystal; *E*_A_ = *E*_B_ is the energy of the isolated BTF molecule. Geometries of the dimers and isolated molecules were taken from the X-ray data.

For a comparison of the crystal packing, we use experimental structures. For salt **1**, a major part of the disorder was used.

## 3. Results and Discussion

### 3.1. Choice of Co-Former

Rational co-crystal design is usually based on the logical and meaningful idea that some particular intermolecular interactions would be energetically favorable and can connect different molecules leading to the formation of the co-crystal [3,66,67]. This idea can be confirmed by the formation of a co-crystal and become experimental evidence, or it may turn out to be unsuitable in some particular cases due to unaccounted synthon competition [68,69]. It is also evident that the stronger intermolecular forces can occur between co-formers, the higher would be the likelihood of the formation of such interactions. Plenty of H-bonded co-crystals and solvates (hydrates) were described [18,70,71,72,73]. Energetic materials, in most cases, are composed of molecules that do not form any strong H-bonds. Moreover, it is likely that strong hydrogen bonds do not contribute to the tight crystal packing [74,75,76] that is so necessary for high energetic materials. Therefore, the co-crystallization strategy of energetic compounds should be based on weaker intermolecular interactions such as, for instance, π…π stacking or O(N)…π interactions that somewhat reduce the prediction ability.

**Figure 1:**
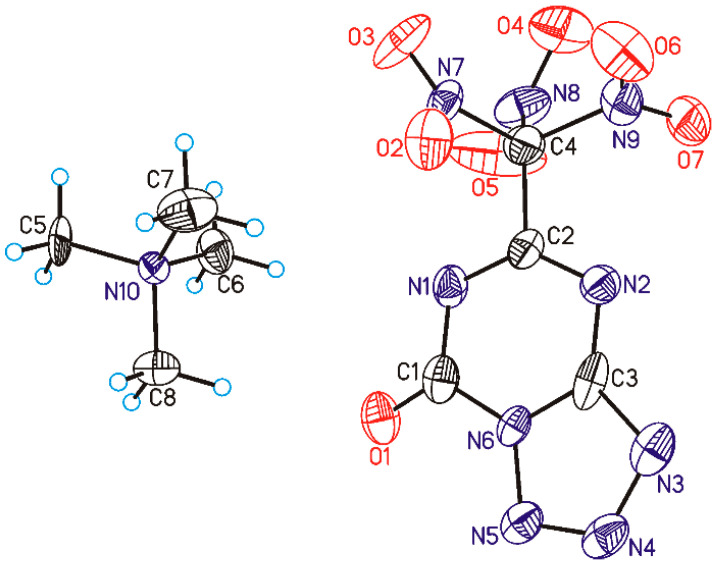
General view of the structure of salt **1** showing atomic numbering. Thermal ellipsoids are given at 50% probability level. Only the first independent molecule A (with unprimed numbering) is shown.

**Figure 2:**
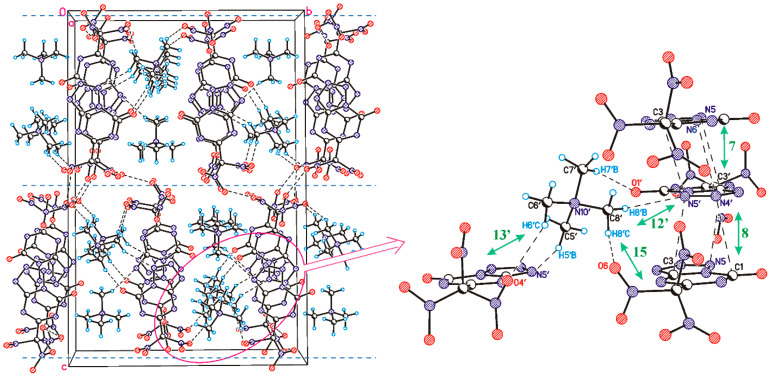
Crystal packing fragment of salt **1**. (**Left**) blue dashed lines indicate interlayer boundaries. (**Right**) shortest intermolecular contacts are shown by black dashed lines; the numbers at green arrows correspond to the numbers in Table 1.

In view of the above, we started from the investigation of the system of intermolecular interactions in the crystal of salt **1** in order to understand the preferential types of intermolecular bonding of the anion and cation. An asymmetric unit cell of salt **1** contains two anions (A and A′) and two cations (C, C′). The molecular and crystal structures are depicted in Figure 1 and Figure 2, while the closest environment and types of intermolecular interactions along with their energies are collected in Table 1 (for more details, see Tables S7–S10 in the Supplementary Materials).

In the crystal, anions are bonded to each other by π…π stacking interactions (entries 7, 8 in Table 1). It should be noted that those interactions link two symmetrically independent anions (A, A′) while A…A and A′…A′ interactions are due to weaker O…O shortened contacts between nitro groups (entries 1–4, 1′–4′ in Table 1). As expected, the tetramethylammonium cation forms C-H…O(N) hydrogen bonds with anions (entries 9–16, 9′–15′ in Table 1). Each C-H…O(N) bond is relatively weak, but due to multiple H-bonded connections, the total pair energy is comparable to the energy of π…π stacking interactions. By π…π stacking interactions and hydrogen bonds, molecules are assembled into the layers (of *c*/2 thickness) parallel to the *ab* plane. Some additional stabilization of the layers is from cation…cation interactions (entries 1CC′, 2CC′ in Table 1). The interlayer interaction is provided by weaker O…O contacts between nitro groups. Based on the data in Table 1, the energies for stacking interaction, hydrogen bonds, and NO_2_…NO_2_ interactions can be estimated to be equal to 6.8, 23.1, and 11.6 kcal/mol, respectively. At the same time, the anion surface involved in stacking interactions is 30.6 Å^2^ that corresponds to 13.1% of the total surface (estimated as average over both symmetrically independent anions, Hirshfeld surface definition is used). The other 204.6 Å^2^ of the anion surface are distributed between hydrogen bonds (55.6%) and NO_2_…NO_2_ interactions (31.3%) (see Figure S3 in the Supplementary Materials for a view of Hirshfeld surfaces).

We can merely search for a co-former based on the ability of salt **1** to form C-H…O(N) hydrogen bonds, and expect that a co-former molecule will be more attractive for cation to bind it. At the same time, it is known (and our results below are in agreement with this) that the energy of π…π stacking interaction is usually underestimated by the EML correlation due to flat potential energy surfaces, low values of the gradient of ρ(r) and overall instability of bond critical points between interacting atoms of the π systems. With this respect, anion…anion interactions observed in the crystal of salt might appear to be the strongest. Therefore, it seems reasonable to take into account those types of interactions that the anion wants to form. It was mentioned above that the anion is involved in stacking interactions. At the same time, being an electron-withdrawing system, it can form anion…π interactions both by its exocyclic oxygen atom and by lone pairs (LP) of nitrogen atoms.

Anion…π contacts are not observed in the crystal structure of salt **1** since the only available π-system in this crystal is obviously electron-rich. However, for relatively strong interaction between the anion and the π-system, the latter must be electron deficient or at least neutral, or at least slightly electron excessive [77]. According to the discussion above, we can indicate several requirements to a co-former for salt **1** listed below.

(1) It must be an energetic compound;

(2) It must contain electron deficient π-system capable of participating in anion…π and π…π stacking interactions;

(3) Since the tetramethylammonium cation is capable of forming C-H…O(N) bond, a potential co-former should contain atoms that supply LP for this.

**Figure 3:**
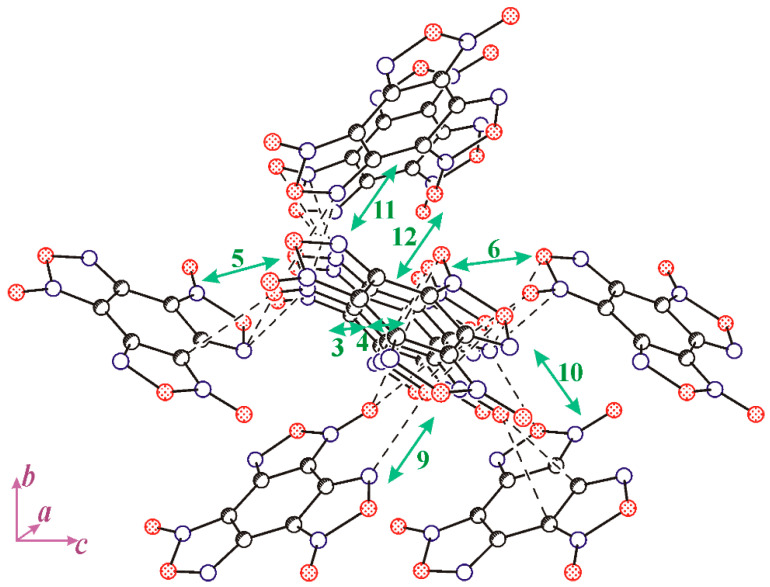
Crystal packing fragment of BTF. The shortest intermolecular contacts are shown by black dashed lines; the numbers at green arrows correspond to the numbers in Table 2.

Benzotrifuroxan (BTF) [37,40] (Figure 3) as a powerful secondary explosive meets these criteria. It is the electron-deficient system with three electron-rich areas located on the outer surface and associated with exocyclic oxygen atoms. BTF is an octopolar molecule with nearly zero total dipole moment, but it can be viewed as consisting of three polar moieties with dipole moments oriented towards the center of the molecule. Consequently, participation of BTF in π…π stacking interactions (taking into account its planar geometry) can be expected as well as in O…π interactions due to electron-rich exocyclic oxygen atoms and electron-deficient center of the molecule. Oxygen and nitrogen atoms of BTF are good LP donor and can participate in hydrogen bonding.

By using Cambridge Structural Database (CSD), one can easily investigate the entire history of BTF itself and its co-crystallization [70]. In many cases, co-crystallization was stabilized by O…π and π…π stacking interactions as well as by C-H…O hydrogen bonds [78,79,80].

Stabilization of the crystal structure of pure BTF was dominated by the π…π stacking and O…π intermolecular interactions (Table 2, more details are provided in Table S11 in the Supplementary Materials). Since BTF is the neutral molecule, we estimated its intermolecular interaction energy both using EML correlation and by calculation of dimeric associates. Both estimations are provided in Table 2. It is seen that some weak interactions are slightly overestimated by the EML correlation while (what is more important) the strong π…π stacking and O…π interactions are significantly underestimated (entries 5, 6, 9, 10 in Table 2). We have already mentioned above the fact that the energies obtained from EML correlation might underestimate such types of noncovalent interactions, and we will keep this in mind when describing the results of co-crystallization in the next section.

Taking all the above into account, we expect that the co-crystallization of BTF and salt **1** will result in a co-crystal formed by means of anion…π and/or π…π stacking interactions between the BTF molecule and the anion and by C-H…O(N) hydrogen bonds. On the other hand, it is also possible that (i) co-crystal will be formed by C-H…O and/or by weak O…O interactions between cation and BTF and between anion and BTF, respectively, while O(N)…π and π…π interactions will be observed between anions and between BTF molecules; (ii) co-crystal will not be formed.

### 3.2. The Structure and Physical Properties of Co-Crystal **3**

**Figure 4:**
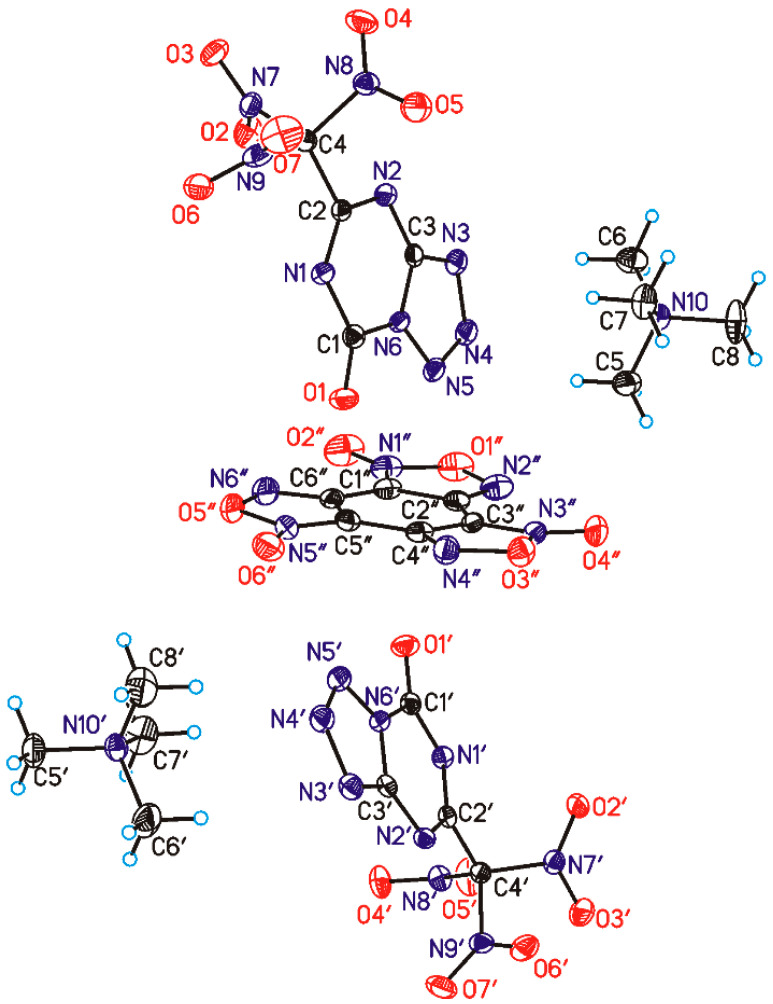
Unit cell content of the co-crystal **3** showing atomic numbering. Thermal ellipsoids are drawn at a 50% probability level.

The X-ray quality yellow–orange crystals of the co-crystal **3** were grown from the slow evaporation of ethanol solution of a mixture of salt **1** and BTF. At first, we defined unit cell parameters that allowed us to assume the formation of a new crystal structure. Single-crystal X-ray diffraction study has revealed a co-crystal formation with the co-former ratio of 1:2 (BTF:salt). An asymmetric unit cell contains one BTF molecule (M), two anions (A, A′), and two cations (C, C′) (Figure 4).

**Figure 5:**
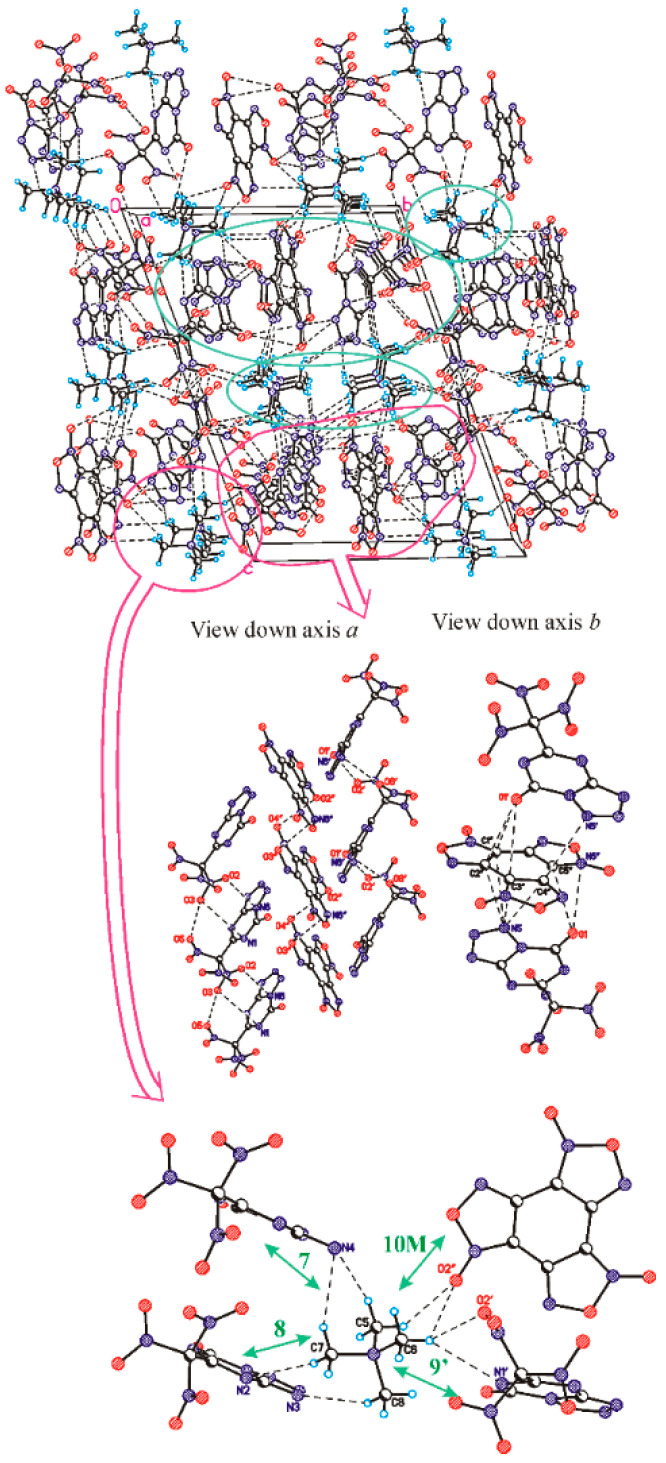
Crystal packing fragment of the co-crystal **3**. (**Top**) green ovals show three types of columns along axis *a*. (**Centre**) the O(N)…π and π…π stacking interactions between BTF and anions. (**Bottom**) the closest environment of the cation C, only the strongest interactions are shown; the numbers at green arrows correspond to the numbers in Table 3.

Even a quick glance at the crystal packing shown in Figure 5 allows us to realize that (i) co-crystal formation occurs due to the O(N)…π interactions between anion and BTF as well as due to the C-H…O hydrogen bonds that are in partial agreement with our expectations; (ii) the crystal structure of the adduct **3** and the environment of the structural units in it are significantly different from that of the original co-formers.

In the co-crystal, BTF molecules act as an electron-deficient π-system for the formation of the O(N)…π interactions with anions. Both sides of the planar BTF molecule are involved in O(N)…π interactions with two anions that probably determine the observed ratio of the co-formers. The geometry of the anions differs from that of the co-former (see Table S1 in the Supplementary Materials) can be due to the different mutual arrangement of structural units in the co-crystal and some flexibility of the trinitromethyl group [81,82]. Cations are extensively involved in the C-H…O(N) hydrogen bonding and make a significant contribution to the crystal packing stabilization (Table 3, for more details, see Tables S12–S16 in the Supplementary Materials).

The crystal structure of **3** can be formally described as built up of columns along axis *a* (green ovals on the top of Figure 5). Three types of columns can be detected. The biggest oval corresponds to the column consisting of anions and BTF molecules linked to each other by the O(N)…π (between anion and BTF), by weak O…π (between anions), and by weak π…π stacking interactions (between BTF molecules). These columns are linked to each other by the O…O contacts between nitro groups. The smallest oval shows the column of cations C, while the remaining oval shows cations C′. Each cation simultaneously interacts with both anions and BTF molecules (Table 3, bottom of Figure 5). However, contrary to salt **1**, the AIM analysis of the electron density of the co-crystal did not reveal any BCPs between cations.

From Table 3, it is also seen that some pair energies for C-H…O bonded structural units (entries 7, 8, 12, 9′, 10′, 11′, 12 M in Table 3) appear to be somewhat higher than the energy of the anion…BTF pair linked by O(N)…π interaction (entries 13, 13′ in Table 3). Here we should recall the above observation that the strong O(N)…π and π…π stacking interactions can be underestimated by the EML formula. Therefore we believe that the O(N)…π interactions are the most important and can be responsible for co-crystal formation.

The above description of the crystal packing of **3** demonstrates quite pronounced rearrangement of the structural units after co-crystallization. At the same time, it is useful to look at the changes in each structural unit separately.

We will consider this from two different points of view. The second and third columns of Table 4 show the energies of structural units in the co-formers and co-crystal (estimated as the sum of all pair interactions formed by a structural unit from Table 1, Table 2 and Table 3). Despite significant changes in the crystal packing, the energies of each unit are still nearly unchanged. The maximum difference does not exceed 3 kcal/mol per structural unit, while the difference in the lattice energies before and after co-crystallization (E_latt_(co-crystal) − E_latt_(BTF) − E_latt_(salt)) is equal to 1.35 kcal/mol, which means that co-crystal formation is less energetically favorable, but the difference is very small. A similar trend is observed for the volumes occupied by units in the co-formers and co-crystal (fourth and fifth columns in Table 4). Using these volumes, we can estimate densification (Δ_OED_ criterion) of each unit before and after co-crystallization, which are also very close (only slight decrease in density and intermolecular energy of structural units is observed upon co-crystallization). It means that all structural units are felt in the same way both in the co-former and in the co-crystal. It can be concluded that salt **1** and BTF can be viewed as an ideal pair for co-crystallization.

The conversion of salt **1** to the co-crystal with BTF increases the density, oxygen coefficient, and enthalpy of formation of the final product, which improves explosive performance and enhances the initiating ability. As can be seen in Table 5, the co-crystal **3** exhibits a superior detonation velocity, detonation pressure, and heat of explosion compared to starting salt **1** that is explained by the inclusion of a powerful BTF unit.

To make a relative comparison of thermal stability and sensitivity, these parameters were measured for the pure co-formers, their ordinary mixture and co-crystalline material, as well as for benchmark metal-free primary explosive tetrazene [90]. These samples were analyzed by differential scanning calorimetry (DSC) and the data are summarized in Table 6. When heated using a 8 °C/min ramp rate, co-crystalline material melted at 153 °C and began to decompose at 167.2 °C, while discrete mixture of salt **1** with BTF (2:1) had a melting point at 157 °C and began to decompose at 167.6 °C. The temperatures of their maximum decomposition were also close. Pure salt **1** had a slightly higher melting point and thermal stability than the co-crystal that can be related to a slight decrease in density and energy of structural units mentioned above. At the same time, both salt **1** and co-crystal were more thermostable than tetrazene.

The sensitivity to impact of all materials was tested using a drop-hammer style instrument K-44-1M designed for handling primary explosives. BTF does not explode when tested on the K-44-1M, while the PETN (pentaerythritol tetranitrate) at a maximum height (50 cm) detonated in only one of 25 trials. Table 6 compares the impact energies, *E*_im_, causing detonation, for the co-formers, their ordinary mixture, and the co-crystal.

For the benchmark primary explosive tetrazene, the *E*_im_ was determined to be 0.3 J, while for salt **1**, a slightly lower sensitivity was found, 0.39 J. Both co-crystallization and simple mixing of BTF with salt **1** (keeping the same molar ratio) lead to a decrease in the impact sensitivity (0.69 J for the co-crystal of 0.6 J for the mixture) compared to the initial salt **1**. Here BTF acts as a phlegmatizer for salt **1**. It should be noted that the observed decrease in sensitivity is relatively small, and the co-crystal **3** is still the primary explosive.

The most interesting fact from the above paragraph and Table 6 is that both ordinary mixture of BTF and salt and their co-crystal have nearly the same sensitivity and thermal stability, which means that there is no difference for structural units whether they are in the co-former or in the co-crystal. This confirms our conclusion on an ideal pair for co-crystallization.

## 4. Conclusions

Recently synthesized tetramethylammonium salt of 7-oxo-5-(trinitromethyl)-4,5,6,7-tetrahydrotetrazolo[1,5-a][1,3,5]triazin-5-ide is the green primary explosive, and is characterized by acceptable sensitivity, but has a relatively low density and energetic performance. In this work, we made an attempt to improve those properties of the salt by its co-crystallization with some appropriate co-former. Detailed crystal packing analysis of the salt allowed to establish the preferential intermolecular interactions for the anion (O(N)…π and π…π stacking interactions) and cation (C-H…O hydrogen bonds). Based on that, high energetic BTF compound, which is able to participate in all those interactions was chosen as a co-former. Stabilization of the crystal structure of the obtained co-crystal was dominated by the anion…π interactions between BTF and anions and by the C-H…O hydrogen bonds cation…BTF cation…anion that was in partial accordance with our expectations. In comparison to the initial salt, the co-crystal has a higher density and better energetic performance due to the inclusion of BTF molecule. At the same time, the sensitivity and thermal stability remained at the same level. Comparison of the crystal structures of all three compounds has revealed significant changes in the crystal packing upon co-crystallization. At the same time, types and energies of intermolecular interactions are still nearly unchanged, and the density of the co-crystal is the nearly additive product of the co-formers. It means that there are no new strong intermolecular interactions formed in the co-crystal, and we believe that minor changes in sensitivity and thermostability are closely related to this fact. Our results demonstrate that with an appropriate choice of co-formers, the energetic properties can be tuned, leaving intact (to some extent) sensitivity and thermal stability, which should be taken into account when developing new high-energetic materials in the future.

## Figures and Tables

**Table 1:** Types of intermolecular interactions and their energies (kcal/mol) obtained at PBE0-D3/POB-TZVP level of theory using EML correlation in the crystal structure of salt **1** ^a^.

Neighbour	Symmetry Code	Type of Interaction	Energy	Molecular Pair
Anion A				
1	−1 + x, y, z	NO_2_…NO_2_	−1.66	A…A
2	1 + x, y, z	NO_2_…NO_2_	−1.66	A…A
3	1 − x, 1 − y, 1 − z	NO_2_…NO_2_	−1.40	A…A
4	2−x, 1−y, 1−z	NO_2_…NO_2_	−0.54	A…A
5	x, y, z	NO_2_…NO_2_	−4.09	A…A′
6	1 + x, y, z	NO_2_…NO_2_	−0.68	A…A′
7	−1/2 + x, 1.5 − y, z − 1/2	π…π stack	−2.71	A…A′
8	1/2 + x, 1.5 − y, −1/2 + z	π…π stack	−4.09	A…A′
9	−1 + x, y, z	C–H…O	−3.02	A…C
10	x, y, z	C–H…O(N)	−4.46	A…C
11	1.5 − x, ½ + y, 1/2 − z	C–H…N	−1.83	A…C
12	1 − x, 1 − y, 1 − z	C–H…O	−1.01	A…C
13	2 − x, 1 − y, 1 − z	C–H…O	−3.52	A…C
14	x, y, z	C–H…O(N)	−2.49	A…C′
15	1.5 − x, 1/2 + y, 1/2 − z	C–H…O(N)	−3.51	A…C′
16	1/2 − x, 1/2 + y, 1/2 − z	C–H…O(N)	−4.10	A…C′
Anion A′				
1′	−1 + x, y, z	NO_2_…NO_2_	−2.66	A′…A′
2′	1 + x, y, z	NO_2_…NO_2_	−2.66	A′…A′
3′	−x, 2 − y, 1 − z	NO_2_…NO_2_	−1.03	A′…A′
4′	1 − x, 2 − y, 1 − z	NO_2_…NO_2_	−2.04	A′…A′
9′	1 − x, 1 − y, 1 − z	C–H…O(N)	−4.17	A′…C
10′	2 − x, 1 − y, 1 − z	C–H…O(N)	−2.32	A′…C
11′	−1/2 + x, 1.5 − y, ½ + z	C–H…N	−2.60	A′…C
12′	1 − x, 1 − y, 1 − z	C–H…O(N)	−4.84	A′…C′
13′	−1/2 + x, 1.5 − y, ½ + z	C–H…O(N)	−4.03	A′…C′
14′	1/2 + x, 1.5 − y, ½ + z	C–H…O(N)	−3.91	A′…C′
15′	1/2 − x, 1/2 + y, 1/2 − z	C–H…O	−0.35	A′…C′
Cation…Cation				
1CC′	1 + x, y, z	vdW	−1.19	C…C′
2CC′	x, y, z	vdW	−0.42	C…C′

^a^ Letters A and C stand for the first independent anion and cation (unprimed), A′ and C′ stand for the second anion and cation (primed), respectively. The same abbreviations are used in Table 3; Entries 5′–8′ are symmetrically equivalent to those of 5–8 and are, therefore, omitted.

**Table 2:** Energies (kcal/mol) of intermolecular interactions in the crystal structure of BTF.

Neighbour	Symmetry Code	Type of Interaction	Energy ^a^	Energy ^b^
Anion A				
1	−1 + x, y, −1 + z	vdW	−1.30	−0.32
2	1 + x, y, 1 + z	vdW	−1.30	−0.32
3	−1 + x, y, z	π…π stack	−1.62	−3.67
4	1 + x, y, z	π…π stack	−1.62	−3.67
5	x, y, −1 + z	π…π stack	−3.77	−5.73
6	x, y, 1 + z	π…π stack	−3.77	−5.73
7	−x, −y, −1/2 + z	vdW	−1.98	−0.24
8	−x, −y, 1/2 + z	vdW	−1.98	−0.24
9	1 − x, −y, −1/2 + z	O(N)…π	−3.97	−5.62
10	1 − x, −y, 1/2 + z	O(N)…π	−3.97	−5.62
11	−1/2 + x, 1/2 − y, z	O(N)…π	−3.93	−3.71
12	1/2 + x, 1/2 − y, z	O(N)…π	3.93	−3.71
13	−1/2 + x, 1/2 − y, −1 + z	vdW	−0.4	−0.49
14	1/2 + x, 1/2 − y, 1 + z	vdW	−0.4	−0.49

^a^ Obtained from the EML correlation at PBE0-D3/POB-TZVP level of theory; ^b^ obtained from the *E*_int_ = *E*_AB_ − *E*_A_ − *E*_B_ formula at M052X/6-311G(df,pd) level of theory.

**Table 3:** Types of intermolecular interactions and their energies (kcal/mol) obtained at PBE0-D3/POB-TZVP level of theory using EML correlation in the crystal structure of the co-crystal **3**.

Neighbour	Symmetry Code	Type of Interaction	Energy	Molecular Pair
Anion A				
1	−1 + x, y, z	weak O…π	−1.86	A…A
2	1 + x, y, z	weak O…π	−1.86	A…A
3	1 − x, 2 − y, −z	NO_2_…NO_2_	−2.48	A…A
4	−1 + x, 1 + y, z	NO_2_…NO_2_	−2.48	A…A
5	2 + x, −1 + y, z	NO_2_…NO_2_/O…π	−2.68	A…A′
6	2 + x, −1 + y, z	NO_2_…NO_2_	−2.27	A…A′
7	x, y, z	C–H…N	−4.51	A…C
8	1 + x, y, z	C–H…O(N)	−4.66	A…C
9	x, −1 + y, z	C–H…O	−2.07	A…C′
10	1 + x, −1 + y, z	C–H…O	−3.45	A…C′
11	−x, 1 − y, 1 − z	C–H…O	−0.82	A…C′
12	1 − x, 1 − y, 1 − z	C–H…O(N)	−5.13	A…C′
13	x, y, z	O(N)…π	−3.95	A…M
14	1 + x, y, z	weak O…π	−2.10	A…M
Anion A′ ^a^				
1′	−1 + x, y, z	weak O…π	−1.83	A′…A′
2′	1 + x, y, z	weak O…π	−1.83	A′…A′
3′	1 − x, 2 − y, −z	NO_2_…NO_2_	−2.21	A′…A′
6′	−1 + x, 1 + y, z	C–H…O	−1.82	A′…C
7′	x, 1 + y, z	C–H…O	−2.40	A′…C
8′	−1 − x, 1 − y, −z	C–H…O	−2.21	A′…C
9′	−x, 1 − y, −z	C–H…O(N)	−4.06	A′…C
10′	−1 + x, y, z	C–H…O(N)	−4.78	A′…C′
11′	x, y, z	C–H…N	−4.14	A′…C′
12′	−x, 1 − y, 1 − z	C–H…N	−1.29	A′…C′
13′	x, y, z	O(N)…π	−3.82	A′…M
14′	−1 + x, y, z	O(N)…π	−2.69	A′…M
BTF molecule (M) ^b^				
1 M	−1 + x, y, z	π…π stacking	−2.67	M…M
2 M	1 + x, y, z	π…π stacking	−2.67	M…M
3 M	1 − x, 1 − y, −z	vdW	−0.46	M…M
8 M	x, y, z	C–H…O(N)	−2.41	M…C
9 M	−x, 1 − y, −z	C–H…O(N)	−1.77	M…C
10 M	1 − x, 1 − y, −z	C–H…O	−3.14	M…C
11 M	x, y, z	C–H…O	−0.50	M…C′
12 M	−x, 1 − y, 1 − z	C–H…O(N)	−4.52	M…C′
13 M	1 − x, 1 − y, 1 − z	C–H…O	−0.50	M…C′

^a^ Entries 4′, 5′ are symmetrically equivalent to those 5, 6 and, therefore, omitted; ^b^ entries 4 M−7 M are symmetrically equivalent to those 13,14,13′,14′ and are therefore, omitted.

**Table 4:** Energies (kcal/mol), volumes (Å^3^), and Δ_OED_ criteria (g/cm^3^) of structural units of compounds 1–3 in their individual form (co-former) and in the co-crystal obtained from periodic calculation at PBE0-D3/POB-TZVP level of theory.

Structural Unit	Energy ^a^		Volume		Δ_OED_ Criterion	
	Co-Former	Co-Crystal	Co-Former	Co-Crystal	Co-Former	Co-Crystal
Anion_1	−40.8	−38.1	251.27	254.27	0.351	0.329
Anion_2	−42.2	−40.3	250.72	253.29	0.355	0.336
Cation_1	−24.6	−26.9	124.85	124.58	0.167	0.169
Cation_2	−24.8	−27.2	123.42	122.92	0.178	0.182
BTF	−34.0	−31.2	214.59	220.82	0.373	0.318

^a^ Total energy of salt **1** is (−40.8 − 42.2 − 24.6 − 24.8) = −132.4 kcal/mol; total energy of BTF is −34.0 kcal/mol; total energy of co-crystal is (−38.1 − 40.3 − 26.9 − 27.2 − 31.2) = −163.7 kcal/mol. The lattice energies (E_latt_) are calculated from those values by dividing by 2, and are equal to −66.2, −17.0, −81.85 for salt, BTF and co-crystal, respectively.

**Table 5:** Physical and calculated energetic properties of co-crystal **3** compared with those of co-formers **1** and **2**.

	Salt 1	BTF 2	Co-Cystal 3
Formula	C_8_H_12_N_10_O_7_	C_6_N_6_O_6_	C_22_H_24_N_26_O_20_
M_W_	360.25	252.1	972.6
α ^[a]^	0.318	0.5	0.357
ρ [g⋅cm^−3^] ^[b]^	1.543	1.898	1.594
ΔH_f_^o^ (*s*) [kJ mol^−1^] (kJ g^−1^) ^[c]^	−157.4 (−0.44)	+580.7 [83] (+2.30)	+245.2 (+0.25)
*D* [m s^−1^] ^[d]^	6740	8740	7200
*P*_C-J_ [GPa] ^[e]^	17.0	34.0	20.0
*Q_expl_* [kJ kg^−1^] ^[f]^	3071.1	6444.6	3861.8

[a] Oxygen coefficient. For a compound with the molecular formula of C_x_H_y_Cl_v_N_w_O_z_, α = (z + v/2)/(2x + y/2). A compound with α > 1 is an oxidizer. [b] Density at 298 K obtained from X-ray analysis. [c] The enthalpy of formation for the solid-state calculated by the additive method [84,85,86,87]. [d] Detonation velocity at maximal density (at *ca* 25 °C). [e] Detonation pressure. [f] Heat of explosion. The explosive performance was predicted using the method developed by Smirnov et al. [88,89].

**Table 6:** Comparison of the sensitivity properties.

Energetic Material	T_m_, ^[a]^ °C	T_onset/peak_, ^[b]^ °C	h_100%_, ^[c]^ cm	*E*_im_, ^[d]^ J
BTF 2	202	230.8/254.8	ND	-
Salt 1	166	177.5/195.2	13	0.39
Co-crystal	153	167.2/189.8	24	0.69
Mixture of 1 with 2 (2:1)	157	167.6/190.3	20	0.6
tetrazene		135/153	10	0.3

[a] Melting point. [b] DSC, decomposition temperature at 10 °C heating ramp rate. [c] Instrument type K-44-1M, 100% drop height, 0.307 kg (Russian instrument for testing primary explosive) [22,36]. [d] Impact energy. ND, no detonation.

## Supplementary Materials

The following are available online. Table S1. Torsion angles of anion defining its conformation in the co-crystal and salt **1**. Figure S1. Overlay of the atomic positions in the experimental structure of the co-crystal (blue) and PBE0-D3/POB-TZVP optimized structure (red). Figure S2. Overlay of the atomic positions in the experimental structure of the salt **1** (major disordered component only, blue) and PBE0-D3/POB-TZVP optimized structure (red). Table S2. PBE0-D3/POB-TZVP optimized atomic coordinates for the BTF crystal. Table S3. PBE0-D3/POB-TZVP optimized atomic coordinates for the salt **1** for major part of the disorder (1st part). Table S4. PBE0-D3/POB-TZVP optimized atomic coordinates for the salt **1** for minor part of the disorder (2nd part). Table S5. PBE0-D3/POB-TZVP optimized atomic coordinates for the co-crystal **3**. Table S6. Volumes (Å3) of structural units obtained using cluster approach and those based on periodic calculation for the co-formers (1, 2) and the co-crystal (**3**).a. Figure S3. Hirshfeld surfaces for anions, cations and BTF molecule in the co formers and co-crystal. Table S7. Pair intermolecular interaction energies (kcal/mol) and shortened contacts (Å) of anion A (unprimed) with its closest environment for salt **1**. Table S8. Pair intermolecular interaction energies (kcal/mol) and shortened contacts (Å) of anion A′ (primed) with its closest environment for salt **1**. Table S9. Pair intermolecular interaction energies (kcal/mol) and shortened contacts (Å) of cation C (unprimed) with its closest environment for salt **1**. Table S10. Pair intermolecular interaction energies (kcal/mol) and shortened contacts (Å) of cation C′ (primed) with its closest environment for salt **1**. Table S11. Pair intermolecular interaction energies (kcal/mol) and shortened contacts (Å) of molecule of BTF with its closest environment in the crystal of BTF **2**. Table S12. Pair intermolecular interaction energies (kcal/mol) and shortened contacts (Å) of anion A (unprimed) with its closest environment for the co-crystal **3**. Table S13. Pair intermolecular interaction energies (kcal/mol) and shortened contacts (Å) of anion A′ (primed) with its closest environment in the co-crystal **3**. Table S14. Pair intermolecular interaction energies (kcal/mol) and shortened contacts (Å) of cation C (unprimed) with its closest environment in the co-crystal **3**. Table S15. Pair intermolecular interaction energies (kcal/mol) and shortened contacts (Å) of cation C′ (primed) with its closest environment in the co-crystal **3**. Table S16. Pair intermolecular interaction energies (kcal/mol) and shortened contacts (Å) of BTF molecule (M) with its closest environment in the co-crystal **3** [47,91,92,93].

## Sample Availability

Samples of the compounds **1**, **2**, **3** are available from the authors.
