# Supplementary material for: Energetic Co-Crystal of a Primary Metal-Free Explosive with BTF. Ideal Pair for Co-Crystallization
Source: Molecules. 2021 Dec 9;26(24):7452. doi: 10.3390/molecules26247452 (PMC8709047; doi:10.3390/molecules26247452)
Supplement: Supplementary file 1 [file molecules-26-07452-s001.zip › molecules-1493324-supplementary.pdf]

Electronic Supporting Information for article

Energetic co-crystal of a primary metal-free explosive with BTF. Ideal pair for co-crystallization

Kyrill Yu. Suponitsky,[*a] Ivan V. Fedyanin,[a] Valentina A. Karnoukhova,[a] Vladimir A. Zalomlenkov,[b] Alexander A. Gidaspov,[b] Vladimir V. Bakharev,[b] Aleksei B. Sheremetev[c]

[a] *A. N. Nesmeyanov Institute of Organoelement Compounds, Russian Academy of Sciences, Vavilov St. 28, Moscow, 119991, Russia*

[b] *Samara State Technical University, Samara, 443100, Russia*

[c] *N. D. Zelinsky Institute of Organic Chemistry, Russian Academy of Sciences, Leninsky Prosp. 47, Moscow, 119991, Russia*

Refinement of the crystal structure of tetramethylammonium salt of 7-oxo-5-(trinitromethyl)-4,5,6,7-tetrahydrotetrazolo[1,5-a][1,3,5]triazin-5-ide (1)

Single crystals of salt **1** were obtained in the form of thin plates of pale-yellow color. Crystals are characterized by extremely low reflection ability. The structure is significantly disordered. Both trinitromethyl groups and one of cations are disordered over two positions. The ratio of the major and minor parts cannot be refined. It was fitted manually as 0.7:0.3. Minor part can be refined only in isotropic approximation using some number of constraints. All our attempts to obtain crystals of better quality were unsuccesful.

Comparative analysis of calculated and experimental crystal structure

In Table S1, torsion angles of the anion in the structure of salt **1** and in the structure of co-crystal are presented. Angles are given for both experimental and theoretically calculated structures. For salt **1**, both parts of the disorder (experimental and theoretically modelled) are presented.

Table S1. Torsion angles of anion defining its conformation in the co-crystal and salt **1**.

Torsion	Co-crystal **3**		Salt **1** (part 1 (70%))		Salt **1** (part 2 (30%))	
angle	X-ray exp.	DFT	X-ray exp.	DFT	X-ray exp.	DFT
N1-C2-C4-N7	-111.75(18)	-113.2	68.1(9)	62.8	-112.4(16)	-127.0
N1-C2-C4-N8	127.10(17)	125.2	-63.5(11)	-57.8	106.4(16)	111.2
N1-C2-C4-N9	6.0(2)	4.1	177.6(7)	178.1	-8.0(13)	-7.1
C2-C4-N7-O2	-6.1(2)	-3.8	35.2(16)	46.7	-34(3)	-27.4
C2-C4-N7-O3	172.86(16)	174.1	-139.0(11)	-132.5	140(3)	153.8
C2-C4-N8-O4	125.87(18)	124.6	-150.3(15)	-162.5	142(2)	140.2
C2-C4-N8-O5	-52.9(2)	-54.1	22.4(18)	18.1	-27(4)	-39.9
C2-C4-N9-O6	-74.7(2)	-76.5	-110.3(11)	-111.4	111(2)	111.6
C2-C4-N9-O7	105.51(19)	103.0	71.1(10)	68.0	-75(3)	-67.9
N1'-C2'-C4'-N7'	64.4(2)	63.4	93.9(8)	90.1	55.8(13)	63.8
N1'-C2'-C4'-N8'	-56.1(2)	-57.3	-158.9(7)	-153.1	-158.9(7)	-174.8
N1'-C2'-C4'-N9'	-176.23(15)	-177.6	-26.2(10)	-27.3	-58.3(16)	-56.3
C2'-C4'-N7'-O2'	-14.3(2)	-14.5	177.6(10)	178.0	-122(3)	-119.7
C2'-C4'-N7'-O3'	166.24(16)	166.0	-4.7(15)	-1.6	53(3)	56.9
C2'-C4'-N8'-O4'	-48.3(2)	-47.7	-111.7(12)	-88.2	-74.9(14)	-107.6
C2'-C4'-N8'-O5'	129.86(18)	130.7	82.5(10)	87.5	82.5(10)	72.0
C2'-C4'-N9'-O6'	-73.97(19)	-73.1	103.2(12)	100.9	28(5)	10.2
C2'-C4'-N9'-O7'	105.82(19)	107.4	-71(2)	-74.3	-175(2)	-169.2

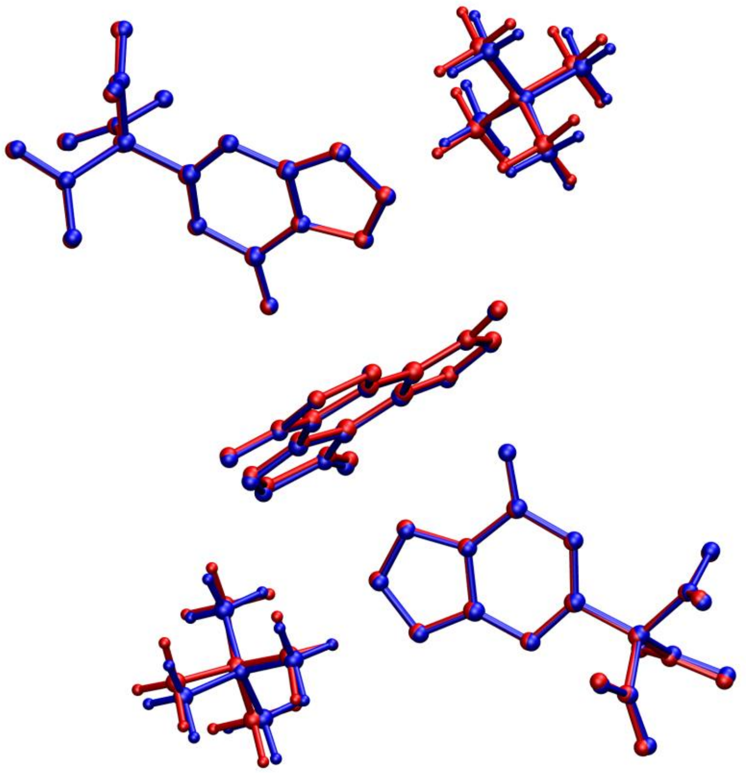

Figure S1. Overlay of the atomic positions in the experimental structure of the co-crystal (blue) and PBE0-D3/POB-TZVP optimized structure (red).

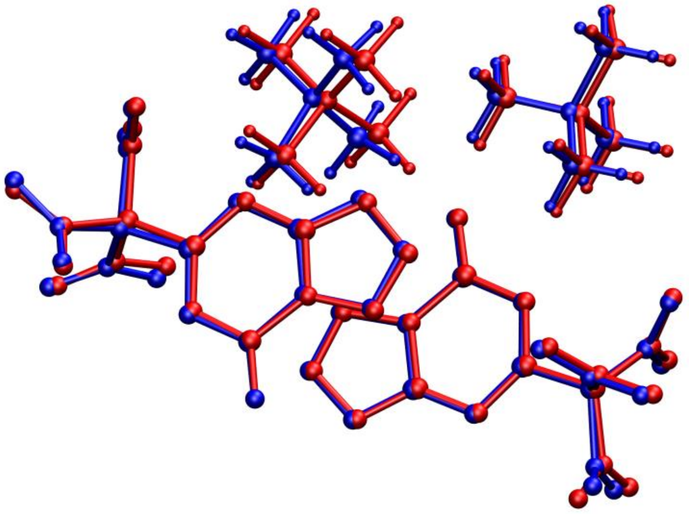

Figure S2. Overlay of the atomic positions in the experimental structure of the salt 1 (major disordered component only, blue) and PBE0-D3/POB-TZVP optimized structure (red).

In four following Tables S2-S5, atomic coordinates for calculated structures are presented in the format of the CIF-file.

Table S2. PBE0-D3/POB-TZVP optimized atomic coordinates for the BTF crystal

```
data_btf-dft

_symmetry_cell_setting           orthorhombic
_symmetry_space_group_name_H-M   'P n a 21'
loop_
_symmetry_equiv_pos_site_id
_symmetry_equiv_pos_as_xyz
1 x,y,z
2 -x,-y,1/2+z
3 1/2-x,1/2+y,1/2+z
4 1/2+x,1/2-y,z
_cell_length_a                   6.8759
_cell_length_b                   19.1923
_cell_length_c                   6.5101
_cell_angle_alpha                90.000
_cell_angle_beta                 90.000
_cell_angle_gamma                90.000
_cell_volume                     859.101
loop_
_atom_site_label
_atom_site_type_symbol
_atom_site_fract_x
_atom_site_fract_y
_atom_site_fract_z
_atom_site_U_iso_or_equiv
_atom_site_thermal_displace_type
O1 O 0.11402 0.02657 0.46881 0.0300 Uiso
O2 O 0.28869 -0.00563 0.7596 0.0300 Uiso
O3 O 0.55613 0.2118 -0.03871 0.0300 Uiso
O4 O 0.26789 0.15636 -0.12012 0.0300 Uiso
O5 O 0.85267 0.1263 0.74133 0.0300 Uiso
O6 O 0.98138 0.20826 0.51568 0.0300 Uiso
N1 N 0.28374 0.02991 0.59955 0.0300 Uiso
N2 N 0.1482 0.06836 0.29616 0.0300 Uiso
N3 N 0.39617 0.16543 0.01091 0.0300 Uiso
N4 N 0.68615 0.2117 0.12627 0.0300 Uiso
N5 N 0.84642 0.1671 0.55316 0.0300 Uiso
N6 N 0.68513 0.08498 0.74854 0.0300 Uiso
C1 C 0.40562 0.0731 0.51153 0.0300 Uiso
C2 C 0.32054 0.09504 0.32365 0.0300 Uiso
C3 C 0.42991 0.14178 0.19708 0.0300 Uiso
C4 C 0.61038 0.17022 0.26236 0.0300 Uiso
C5 C 0.68571 0.14912 0.4576 0.0300 Uiso
C6 C 0.58914 0.0991 0.58142 0.0300 Uiso
```

Table S3. PBE0-D3/POB-TZVP optimized atomic coordinates for the salt 1 for major part of the disorder (1st part)

```
data_salt-1-dft

_symmetry_cell_setting            monoclinic
_symmetry_space_group_name_H-M    'P 21/n'
loop_
_symmetry_equiv_pos_site_id
_symmetry_equiv_pos_as_xyz
1 x,y,z
2 1/2-x,1/2+y,1/2-z
3 -x,-y,-z
4 1/2+x,1/2-y,1/2+z
_cell_length_a                    6.2668
_cell_length_b                    17.9111
_cell_length_c                    26.7510
_cell_angle_alpha                 90.000
_cell_angle_beta                  93.399
_cell_angle_gamma                 90.000
_cell_volume                      2997.39
loop_
_atom_site_label
_atom_site_type_symbol
_atom_site_fract_x
_atom_site_fract_y
_atom_site_fract_z
_atom_site_U_iso_or_equiv
_atom_site_thermal_displace_type
O1 O 0.52296 0.46965 0.30284 0.0300 Uiso
N1 N 0.54637 0.68556 0.34927 0.0300 Uiso
N2 N 0.57848 0.55452 0.36646 0.0300 Uiso
N3 N 0.50763 0.59506 0.28513 0.0300 Uiso
N4 N 0.47365 0.59372 0.23409 0.0300 Uiso
N5 N 0.46247 0.66412 0.22079 0.0300 Uiso
N6 N 0.48698 0.71118 0.2609 0.0300 Uiso
C1 C 0.57607 0.6261 0.37773 0.0300 Uiso
C2 C 0.53574 0.53342 0.31801 0.0300 Uiso
C3 C 0.51441 0.6673 0.30055 0.0300 Uiso
C4 C 0.63776 0.64223 0.43248 0.0300 Uiso
O2 O 0.9919 0.63609 0.41125 0.0300 Uiso
O3 O 0.90859 0.57792 0.48053 0.0300 Uiso
O4 O 0.52535 0.63464 0.51355 0.0300 Uiso
O5 O 0.39376 0.55156 0.45877 0.0300 Uiso
O6 O 0.79348 0.75859 0.45383 0.0300 Uiso
O7 O 0.44125 0.74993 0.44087 0.0300 Uiso
N7 N 0.86696 0.61633 0.44278 0.0300 Uiso
N8 N 0.50529 0.60555 0.47132 0.0300 Uiso
N9 N 0.62511 0.72453 0.44364 0.0300 Uiso
N10' N 0.41488 0.36983 0.16582 0.0300 Uiso
C5' C 0.60548 0.4055 0.19207 0.0300 Uiso
H5'A H 0.55918 0.45983 0.20585 0.0300 Uiso
```

H5'B H 0.72896 0.41159 0.16548 0.0300 Uiso
H5'C H 0.65822 0.36977 0.22326 0.0300 Uiso
C6' C 0.34224 0.41561 0.12152 0.0300 Uiso
H6'A H 0.30368 0.47133 0.13436 0.0300 Uiso
H6'B H 0.20169 0.38926 0.10336 0.0300 Uiso
H6'C H 0.47025 0.41801 0.09583 0.0300 Uiso
C7' C 0.24014 0.36424 0.20106 0.0300 Uiso
H7'A H 0.20131 0.41991 0.21409 0.0300 Uiso
H7'B H 0.29662 0.32868 0.23195 0.0300 Uiso
H7'C H 0.10268 0.33851 0.18125 0.0300 Uiso
C8' C 0.47156 0.29317 0.14953 0.0300 Uiso
H8'A H 0.33249 0.26849 0.12964 0.0300 Uiso
H8'B H 0.51757 0.26017 0.18252 0.0300 Uiso
H8'C H 0.6027 0.29736 0.12496 0.0300 Uiso
C5 C 0.14088 0.29736 0.3902 0.0300 Uiso
H5A H 0.05448 0.24834 0.37605 0.0300 Uiso
H5B H 0.15278 0.29752 0.43091 0.0300 Uiso
H5C H 0.29932 0.29847 0.37564 0.0300 Uiso
C6 C -0.1959 0.36519 0.39174 0.0300 Uiso
H6A H -0.27752 0.3145 0.37882 0.0300 Uiso
H6B H -0.28079 0.41427 0.37725 0.0300 Uiso
H6C H -0.17939 0.36659 0.43241 0.0300 Uiso
C7 C 0.13894 0.43392 0.38946 0.0300 Uiso
H7A H 0.2991 0.43219 0.37601 0.0300 Uiso
H7B H 0.14508 0.4356 0.43012 0.0300 Uiso
H7C H 0.054 0.4821 0.3735 0.0300 Uiso
C8 C 0.00426 0.36447 0.31632 0.0300 Uiso
H8A H -0.07425 0.31316 0.3037 0.0300 Uiso
H8B H 0.16553 0.36726 0.30348 0.0300 Uiso
H8C H -0.08774 0.41282 0.30336 0.0300 Uiso
N10' N 0.02205 0.36519 0.37212 0.0300 Uiso
O1' O 0.96331 0.76447 0.21353 0.0300 Uiso
O5' O 1.1028 0.52886 0.04595 0.0300 Uiso
O2' O 0.53205 0.58241 0.02325 0.0300 Uiso
O3' O 0.47793 0.60078 0.10384 0.0300 Uiso
O4' O 0.80511 0.47393 0.06688 0.0300 Uiso
O6' O 0.74998 0.71439 0.03756 0.0300 Uiso
O7' O 1.0875 0.67726 0.04284 0.0300 Uiso
N7' N 0.58983 0.59447 0.06744 0.0300 Uiso
N9' N 0.89572 0.66973 0.04924 0.0300 Uiso
N1' N 0.92426 0.55265 0.15949 0.0300 Uiso
N2' N 0.90055 0.68491 0.14702 0.0300 Uiso
N3' N 0.96951 0.63774 0.22671 0.0300 Uiso
N4' N 1.00634 0.63478 0.27764 0.0300 Uiso
N5' N 1.01875 0.56348 0.28823 0.0300 Uiso
N6' N 0.9919 0.51982 0.24644 0.0300 Uiso
N8' N 0.92087 0.52898 0.061 0.0300 Uiso
C1' C 0.89168 0.61418 0.13342 0.0300 Uiso
C2' C 0.94534 0.702 0.19615 0.0300 Uiso
C3' C 0.96142 0.56689 0.20858 0.0300 Uiso
C4' C 0.82855 0.602 0.07815 0.0300 Uiso

Table S4. PBE0-D3/POB-TZVP optimized atomic coordinates for the salt 1 for minor part of the disorder (2nd part)

```
data_salt-2-dft

_symmetry_cell_setting           monoclinic
_symmetry_space_group_name_H-M   'P 21/n'
loop_
_symmetry_equiv_pos_site_id
_symmetry_equiv_pos_as_xyz
1 x,y,z
2 1/2-x,1/2+y,1/2-z
3 -x,-y,-z
4 1/2+x,1/2-y,1/2+z
_cell_length_a                   6.2668
_cell_length_b                   17.9111
_cell_length_c                   26.7510
_cell_angle_alpha                90.000
_cell_angle_beta                 93.399
_cell_angle_gamma                90.000
_cell_volume                     2997.39
loop_
_atom_site_label
_atom_site_type_symbol
_atom_site_fract_x
_atom_site_fract_y
_atom_site_fract_z
_atom_site_U_iso_or_equiv
_atom_site_thermal_displace_type
O1 O 0.52316 0.47965 0.2828 0.0300 Uiso
N1 N 0.5857 0.68198 0.35085 0.0300 Uiso
N2 N 0.57675 0.54844 0.35459 0.0300 Uiso
N3 N 0.53139 0.60756 0.27812 0.0300 Uiso
N4 N 0.50055 0.61823 0.22753 0.0300 Uiso
N5 N 0.50369 0.69075 0.22165 0.0300 Uiso
N6 N 0.53569 0.72788 0.2661 0.0300 Uiso
C1 C 0.59309 0.61661 0.37313 0.0300 Uiso
C2 C 0.54215 0.53917 0.30426 0.0300 Uiso
C3 C 0.5523 0.67534 0.30085 0.0300 Uiso
C4 C 0.6456 0.61895 0.42941 0.0300 Uiso
O2A O 0.40645 0.71833 0.4408 0.0300 Uiso
O3A O 0.49431 0.64435 0.50502 0.0300 Uiso
O4A O 0.90208 0.69243 0.47499 0.0300 Uiso
O5A O 1.00234 0.6266 0.41024 0.0300 Uiso
O6A O 0.80881 0.51332 0.46712 0.0300 Uiso
O7A O 0.45832 0.51393 0.44967 0.0300 Uiso
N7A N 0.50057 0.66496 0.46087 0.0300 Uiso
N8A N 0.87025 0.64904 0.43937 0.0300 Uiso
N9A N 0.6386 0.54163 0.45091 0.0300 Uiso
N10 N 0.43913 0.36782 0.16136 0.0300 Uiso
C5" C 0.64877 0.3964 0.18278 0.0300 Uiso
H5"A H 0.75517 0.40277 0.15237 0.0300 Uiso
```

H5"B H 0.62271 0.44967 0.20087 0.0300 Uiso
H5"C H 0.71264 0.35604 0.21008 0.0300 Uiso
C6" C 0.35775 0.41723 0.11976 0.0300 Uiso
H6"A H 0.34396 0.47378 0.13399 0.0300 Uiso
H6"B H 0.46913 0.41572 0.09014 0.0300 Uiso
H6"C H 0.20242 0.39674 0.10564 0.0300 Uiso
C7" C 0.28229 0.36659 0.20117 0.0300 Uiso
H7"A H 0.25726 0.42342 0.21386 0.0300 Uiso
H7"B H 0.34762 0.33181 0.23169 0.0300 Uiso
H7"C H 0.13419 0.34247 0.18533 0.0300 Uiso
C8" C 0.46637 0.29043 0.14226 0.0300 Uiso
H8"A H 0.51338 0.25437 0.17368 0.0300 Uiso
H8"B H 0.58938 0.29133 0.11523 0.0300 Uiso
H8"C H 0.31509 0.27179 0.12459 0.0300 Uiso
C5 C 0.14974 0.31243 0.38234 0.0300 Uiso
H5A H 0.07591 0.26045 0.36931 0.0300 Uiso
H5B H 0.17437 0.31266 0.4229 0.0300 Uiso
H5C H 0.30058 0.31991 0.36485 0.0300 Uiso
C6 C -0.203 0.36648 0.3908 0.0300 Uiso
H6A H -0.27411 0.31344 0.37905 0.0300 Uiso
H6B H -0.30616 0.4127 0.37852 0.0300 Uiso
H6C H -0.17234 0.36759 0.43125 0.0300 Uiso
C7 C 0.10527 0.44738 0.38253 0.0300 Uiso
H7A H 0.25903 0.45184 0.36586 0.0300 Uiso
H7B H 0.1267 0.44827 0.42308 0.0300 Uiso
H7C H 0.00181 0.4926 0.36905 0.0300 Uiso
C8 C -0.03339 0.3748 0.3113 0.0300 Uiso
H8A H -0.10024 0.32114 0.29999 0.0300 Uiso
H8B H 0.11953 0.3839 0.29512 0.0300 Uiso
H8C H -0.14441 0.4194 0.30074 0.0300 Uiso
N10 N 0.00445 0.37519 0.36687 0.0300 Uiso
O1' O -0.01691 0.77229 0.20783 0.0300 Uiso
O5' O 0.06566 0.51507 0.05302 0.0300 Uiso
O2" O -0.42578 0.68955 0.0373 0.0300 Uiso
O3" O -0.50207 0.60474 0.09399 0.0300 Uiso
O4" O -0.28292 0.4928 0.04943 0.0300 Uiso
O6" O 0.09454 0.70434 0.04606 0.0300 Uiso
O7" O -0.07966 0.6345 -0.01123 0.0300 Uiso
N7" N -0.38108 0.6374 0.06636 0.0300 Uiso
N9" N -0.03294 0.65532 0.03228 0.0300 Uiso
N1' N -0.04108 0.56175 0.15144 0.0300 Uiso
N2' N -0.07729 0.69401 0.14076 0.0300 Uiso
N3' N 0.00059 0.64538 0.21963 0.0300 Uiso
N4' N 0.03328 0.64131 0.2706 0.0300 Uiso
N5' N 0.04458 0.56979 0.28056 0.0300 Uiso
N6' N 0.02175 0.52702 0.23828 0.0300 Uiso
N8' N -0.12364 0.53272 0.05647 0.0300 Uiso
C1' C -0.07575 0.62389 0.12605 0.0300 Uiso
C2' C -0.0304 0.71009 0.18986 0.0300 Uiso
C3' C -0.00593 0.57493 0.20079 0.0300 Uiso
C4' C -0.14937 0.61295 0.07145 0.0300 Uiso

Table S5. PBE0-D3/POB-TZVP optimized atomic coordinates for the co-crystal **3**

```
data_cc-dft

_symmetry_cell_setting            triclinic
_symmetry_space_group_name_H-M    'P -1'
loop_
_symmetry_equiv_pos_site_id
_symmetry_equiv_pos_as_xyz
1 x,y,z
2 -x,-y,-z
_cell_length_a                    6.8749
_cell_length_b                    14.8319
_cell_length_c                    20.4613
_cell_angle_alpha                 70.487
_cell_angle_beta                  83.743
_cell_angle_gamma                 87.694
_cell_volume                      1954.83
loop_
_atom_site_label
_atom_site_type_symbol
_atom_site_fract_x
_atom_site_fract_y
_atom_site_fract_z
_atom_site_U_iso_or_equiv
_atom_site_thermal_displace_type
O1 O 0.35385 0.2729 0.36046 0.0300 Uiso
O2 O 1.07228 0.12077 0.2926 0.0300 Uiso
O3 O 1.14314 -0.01186 0.37651 0.0300 Uiso
O4 O 0.88639 -0.10717 0.33039 0.0300 Uiso
O5 O 0.5831 -0.08464 0.37146 0.0300 Uiso
O6 O 0.88354 0.0776 0.45537 0.0300 Uiso
O7 O 0.70358 -0.05323 0.48409 0.0300 Uiso
N1 N 0.58119 0.15369 0.36437 0.0300 Uiso
N2 N 0.67723 0.11317 0.26128 0.0300 Uiso
N3 N 0.52494 0.21514 0.16115 0.0300 Uiso
N4 N 0.3978 0.29 0.15506 0.0300 Uiso
N5 N 0.35169 0.305 0.21421 0.0300 Uiso
N6 N 0.4544 0.23795 0.26112 0.0300 Uiso
N7 N 1.02906 0.04718 0.34211 0.0300 Uiso
N8 N 0.75781 -0.06355 0.35504 0.0300 Uiso
N9 N 0.79726 0.01478 0.44244 0.0300 Uiso
C1 C 0.45534 0.22419 0.33294 0.0300 Uiso
C2 C 0.67829 0.10651 0.32705 0.0300 Uiso
C3 C 0.55941 0.18377 0.22771 0.0300 Uiso
C4 C 0.81332 0.0277 0.36562 0.0300 Uiso
N10 N 0.04969 0.21188 0.05248 0.0300 Uiso
C5 C 0.04995 0.3139 0.04951 0.0300 Uiso
H5A H 0.17613 0.3247 0.07365 0.0300 Uiso
H5B H -0.08495 0.32744 0.07797 0.0300 Uiso
H5C H 0.05839 0.35947 -0.00464 0.0300 Uiso
C6 C 0.23116 0.19059 0.01334 0.0300 Uiso
```

H6A H 0.35617 0.20629 0.03596 0.0300 Uiso
H6B H 0.23329 0.23428 -0.04117 0.0300 Uiso
H6C H 0.23021 0.11505 0.01871 0.0300 Uiso
C7 C 0.04187 0.14801 0.12644 0.0300 Uiso
H7A H 0.17156 0.16068 0.14797 0.0300 Uiso
H7B H 0.03789 0.07431 0.12764 0.0300 Uiso
H7C H -0.08976 0.16379 0.15516 0.0300 Uiso
C8 C -0.12427 0.19592 0.02027 0.0300 Uiso
H8A H -0.11358 0.24334 -0.03362 0.0300 Uiso
H8B H -0.25465 0.2109 0.05024 0.0300 Uiso
H8C H -0.12418 0.12197 0.02199 0.0300 Uiso
O1' O 0.03264 -0.32282 0.16856 0.0300 Uiso
O2' O -0.58313 -0.22187 0.09882 0.0300 Uiso
O3' O -0.77529 -0.09711 0.09527 0.0300 Uiso
O4' O -0.28702 -0.01689 0.16079 0.0300 Uiso
O5' O -0.4213 -0.00129 0.0639 0.0300 Uiso
O6' O -0.80418 -0.16619 0.24168 0.0300 Uiso
O7' O -0.67018 -0.02562 0.22557 0.0300 Uiso
N1' N -0.23933 -0.22644 0.16663 0.0300 Uiso
N2' N -0.42272 -0.23113 0.27436 0.0300 Uiso
N3' N -0.27173 -0.33175 0.37495 0.0300 Uiso
N4' N -0.10591 -0.38621 0.37948 0.0300 Uiso
N5' N -0.01854 -0.38072 0.31809 0.0300 Uiso
N6' N -0.13247 -0.3202 0.27139 0.0300 Uiso
N7' N -0.63673 -0.1519 0.11581 0.0300 Uiso
N8' N -0.40087 -0.04014 0.1266 0.0300 Uiso
N9' N -0.67895 -0.10315 0.21598 0.0300 Uiso
C1' C -0.10318 -0.29097 0.19769 0.0300 Uiso
C2' C -0.38444 -0.2041 0.20639 0.0300 Uiso
C3' C -0.28595 -0.29121 0.3073 0.0300 Uiso
C4' C -0.52256 -0.12766 0.16694 0.0300 Uiso
N10' N 0.20529 -0.29334 0.48388 0.0300 Uiso
C5' C 0.35237 -0.26005 0.51916 0.0300 Uiso
H5'A H 0.49382 -0.29058 0.50858 0.0300 Uiso
H5'B H 0.30804 -0.28411 0.57476 0.0300 Uiso
H5'C H 0.35845 -0.18243 0.49884 0.0300 Uiso
C6' C 0.00683 -0.2569 0.49988 0.0300 Uiso
H6'A H -0.09584 -0.2811 0.47241 0.0300 Uiso
H6'B H 0.01322 -0.17936 0.48205 0.0300 Uiso
H6'C H -0.03427 -0.28444 0.55575 0.0300 Uiso
C7' C 0.2609 -0.25696 0.40721 0.0300 Uiso
H7'A H 0.40748 -0.28235 0.3972 0.0300 Uiso
H7'B H 0.25783 -0.17931 0.38939 0.0300 Uiso
H7'C H 0.15741 -0.28434 0.38214 0.0300 Uiso
C8' C 0.20361 -0.40001 0.50869 0.0300 Uiso
H8'A H 0.34952 -0.4248 0.49628 0.0300 Uiso
H8'B H 0.09787 -0.423 0.48179 0.0300 Uiso
H8'C H 0.16332 -0.42539 0.56459 0.0300 Uiso
O1" O 0.33631 -0.44338 0.04435 0.0300 Uiso
O2" O 0.61965 -0.39845 0.07755 0.0300 Uiso
O3" O -0.203 -0.62881 0.2803 0.0300 Uiso
O4" O -0.23623 -0.60133 0.16392 0.0300 Uiso
O5" O 0.50984 -0.47572 0.31845 0.0300 Uiso

```
O6" O 0.26076 -0.54915 0.40262 0.0300 Uiso
N1" N 0.45471 -0.43424 0.09684 0.0300 Uiso
N2" N 0.15914 -0.48694 0.0786 0.0300 Uiso
N3" N -0.13414 -0.58877 0.20671 0.0300 Uiso
N4" N -0.06471 -0.60788 0.31866 0.0300 Uiso
N5" N 0.32054 -0.52238 0.33992 0.0300 Uiso
N6" N 0.545 -0.44921 0.24559 0.0300 Uiso
C1" C 0.34918 -0.46941 0.15709 0.0300 Uiso
C2" C 0.16967 -0.50146 0.14437 0.0300 Uiso
C3" C 0.03265 -0.54681 0.20327 0.0300 Uiso
C4" C 0.07271 -0.5602 0.27269 0.0300 Uiso
C5" C 0.25242 -0.52357 0.28265 0.0300 Uiso
C6" C 0.39207 -0.47806 0.22578 0.0300 Uiso
```

The Δ_{OED} – based densification approach

According to the approach, it is assumed that upon crystal formation, molecules interact to each other by means of overlap of their electron densities. It means that volume of isolated molecule is larger than that of the molecule in a crystal. Similarly, density of an isolated molecule (d_{mol}) is lower than that in a crystal (d_{cryst}). The latter is the density of the crystal structure obtained from X-ray experiment. In the other words, upon crystal structure formation, molecule is densified. The value of d_{mol} can be estimated by analysis of the electron density of optimized isolated molecule in terms of the AIM theory. It is defined as a ratio of molecular mass per molecular volume (the latter is presented as the sum of atomic volumes)

$$d_{mol} = m_{mol}/V_{mol}\ ; \qquad m_{mol} = M_{mol}/N_A\ ; \qquad V_{mol} = \sum_i V_{at}^{(i)}$$

Here, M_{mol} and m_{mol} are molar and molecular masses, respectively, N_A is Avogadro number, V_{mol} and V_{at} are molecular and atomic volumes, respectively. It is convenient to present d_{mol} in g/cm^3 units. Evidently, the volume and density of any molecular fragment can be calculated in a similar way. For estimation of V_{mol}, isodensity surface of 0.0004 e/a_o^3 (a_o – Bohr radius) was utilized for integration procedure. So estimated molecular volume comprises about 99.8% of all electrons (nearly whole molecule), and charge leakage does not exceed 0.002 e/Å^3 that approximately corresponds to numerical error of integration of calculated electron density. It is evident, that difference between crystal and molecular density can be served as a measure of how pronounced is the overlap of molecular electron densities upon crystal structure formation. Therefore, the Δ_{OED} criterion defined as $\Delta_{OED} = d_{cryst} – d_{mol}$ would characterize a degree of molecular densification and, therefore, tightness of crystal packing. Combined analysis of d_{mol} and Δ_{OED} values would clarify an origin of the observed crystal density.

In the case of one molecule in the asymmetric unit cell, density of a molecule in a crystal is simply equal to crystal density that is obtained from X-ray experiment. For estimation of density of an isolated molecule, its geometry was calculated at the M052X/6-311G(df,pd) level of theory that was succesfully utilized in our recent studies on polynitro compounds. The GAUSSIAN program was used for calculation.[1] The wave functions obtained from calculation of isolated molecules were analyzed in terms of R. Bader "AIM" topological theory[2] using the AIMALL program.[3]

However, the Δ_{OED} – based approach can be applied not only to the whole molecule, but also to any molecular unit or structural unit, if there are more than one structural unit in the asymmetric unit cell. In such case one needs to define a way of estimation of the volume of the unit in a crystal. To do this, one can generate cluster in which the unit under study is surrounded by its environment. The supramolecular clusters are generated in such a way that each atom of the unit has all geometric contacts within the sphere of 5 Å radii. To obtain electron density

distribution is such clusters, lower level of theory, namely, HF/3-21G is used because of huge size of a cluster. AIM partitioning of so obtained electron density can be carried out using the MultiWFN program.[4]

Another way to obtain atomic volumes is based on AIM partitioning of the crystal electron density which can be obtained from accurate X-ray experiment (using multipole refinement) or from periodic calculation. This is more time consuming way, however in the present work (as written in the main text of the article), periodic calculation were carried out in order to estimate energetic properties of the crystals under study. Therefore volumes of structural units discussed in this work were obtained from periodic calculations.

For the sake of comparison, the first way of obtaining of volumes was also used, and the results are shown in Table 6S.

Table S6. Volumes (Å^3) of structural units obtained using cluster approach and those based on periodic calculation for the co-formers (**1**, **2**) and the co-crystal (**3**).[a]

Structural unit	Volume (cluster approach)		Volume (periodic calc.)	
	Co-former	Co-crystal	Co-former	Co-crystal
Anion_1	250.29	255.51	251.27	254.27
Anion_2	249.79	252.71	250.72	253.29
Cation_1	126.23	125.29	124.85	124.58
Cation_2	124.40	124.95	123.42	122.92
BTF	214.78	218.92	214.59	220.82

[a] the results from periodic calculations are presented in Table 4 of the main text of this article

It can be seen that good agreement is observed between both ways of calculation.

Strictly speaking, volume of pure BTF molecule and sums of the volumes of structural units for salt **1** and the co-crystal **3** should be equal to the volumes of asymmetric unit cells. However, due to numerical errors of integration of calculated electron density, some disagreement can be observed. To check this we estimated densities of all three crystals from the results of Table 6S and compared those values with experimentally obtained crystal densities. As shown below, an agreement is excellent.

	salt **1** (g/cm^3)	BTF **2** (g/cm^3)	co-crystal **3** (g/cm^3)
calculated (periodic calc.)	1.595	1.950	1.655
calculated (cluster approach)	1.594	1.949	1.653
experimental	1.597	1.949	1.652

Salt (**1**):

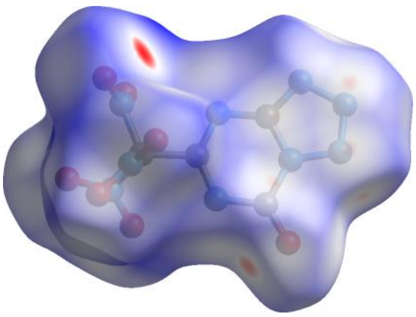

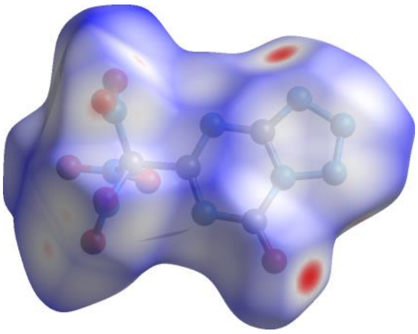

Anion_1 Anion_2

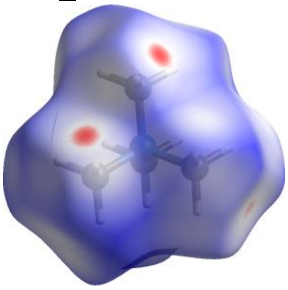

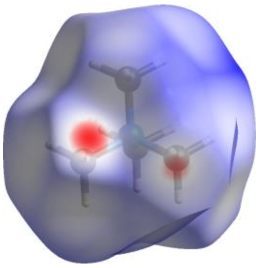

Cation_1 Cation_2

BTF (**2**):

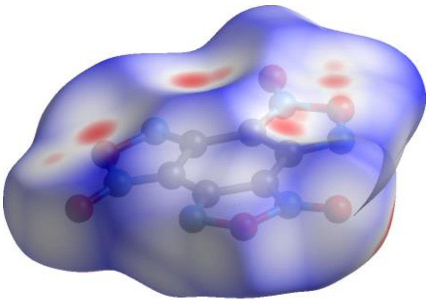

Co-crystal (**3**)

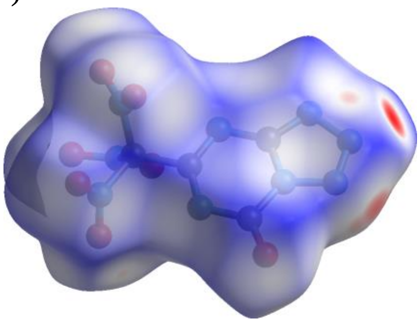

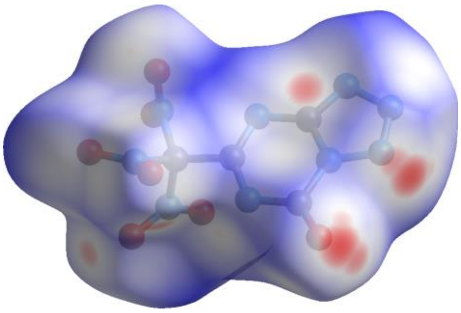

Anion_1 Anion_2

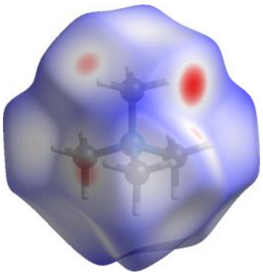

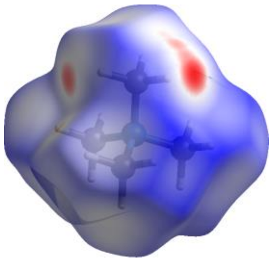

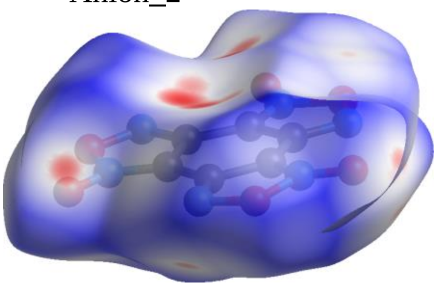

Cation_1 Cation_2 BTF

Figure S3. Hirshfeld surfaces for anions, cations and BTF molecule in the co formers and co-crystal.

Comments to Tables S7-S16

In Tables S7-S16, the atom…atom energies are obtained at PBE0-D3/POB-TZVP level of theory using EML correlation.

Comments to Tables S7-S10

The first column (entry) stands for numbering of the closest neighbours. Due to the disorder, one can potentially observe four ways of unit…unit interaction which will include: 1) major parts of the disorder (p1…p1); 2) minor parts of the disorder (p2…p2); 3) major part with minor part (p1…p2); 4) minor part with major part (p2…p1). All of them are shown in Tables. Major part is denoted as p1, while minor part is denoted as p2. For calculated structure, we have only two ways: 1) major part with major part (p1…p1); 2) minor part with minor part (p2…p2). For estimation of unit…unit energy, we used average sum of p1…p1 and p2…p2 interaction energies taking into account experimentally obtained occupancies (0.7:0.3).

In those cases when undisordered parts of both strucural units are involved into the system of shortened intermolecular contacts, all four ways are equivalent, and only one is presented. In those cases when undisordered part of one strucural unit and disordered part of the other structural unit are involved into the system of shortened intermolecular contacts, only two ways are presented (p1…p1 and p2…p2).

For calculated structure, distances are given only for those atomic pairs between which BCP was localized.

Symmetrically equivalent intermolecular interactions in Tables S2-S5 are marked with the same colors.

Table S7. Pair intermolecular interaction energies (kcal/mol) and shortened contacts (Å) of anion A (unprimed) with its closest environment for salt **1**.

Entry	Symmetry code	Atomic pair		Distance X-ray	Distance calc.	Energy	Type of interaction	Molecular pair
1	-1+x,y,z	O5	O2	2.866	3.142	-0.73	NO_2…NO_2	A…A
		O5	O3	3.130	3.165	-0.68		
		O7	O2	3.823	3.529	-0.25		
1^{p1-p2}	-1+x,y,z	No close contact			–	–	vdW	A…A
1^{p2-p1}	-1+x,y,z	O2A	O2	2.821	–	–	NO_2…NO_2	A…A
1^{p2-p2}	-1+x,y,z	O5A	O2A	2.721	3.088	-0.88	NO_2…NO_2	A…A
		O5A	O7A	4.022	3.605	-0.21		
2	1+x,y,z	O2	O5	2.866	3.142	-0.73	NO_2…NO_2	A…A
		O3	O5	3.130	3.165	-0.68		
		O2	O7	3.823	3.529	-0.25		
2^{p1-p2}	1+x,y,z	O2	O2A	2.821	–	–	NO_2…NO_2	A…A
2^{p2-p1}	-1+x,y,z	No close contact			–	–	vdW	A…A
2^{p2-p2}	-1+x,y,z	O2A	O5A	2.721	3.088	-0.88	NO_2…NO_2	A…A
		O7A	O5A	4.022	3.605	-0.21		
3	1-x,1-y,1-z	O5	O5	3.522	3.115	-0.80	NO_2…NO_2	A…A
		O3	O5	3.654	3.460	-0.30		
		O5	O3	3.654	3.460	-0.30		
3^{p1-p2}	1-x,1-y,1-z	O5	O7A	2.812	–	–	NO_2…NO_2	A…A
3^{p2-p1}	1-x,1-y,1-z	O7A	O5	2.812	–	–	NO_2…NO_2	A…A
3^{p2-p2}	1-x,1-y,1-z	O3A	O7A	2.773	3.091	-0.91	NO_2…NO_2	A…A
		O7A	O3A	2.773	3.091	-0.91		
		O6A	O7A	2.818				
		O7A	O6A	2.818				
		O7A	O7A	2.241	2.758	-2.70		
4	2-x,1-y,1-z	O3	O3	3.242	3.170	-0.54	NO_2…NO_2	A…A
4^{p1-p2}	2-x,1-y,1-z	O3	O6A	2.890	–	–	NO_2…NO_2	A…A
4^{p2-p1}	2-x,1-y,1-z	O6A	O3	2.890	–	–	NO_2…NO_2	A…A
4^{p2-p2}	2-x,1-y,1-z	O6A	O6A	2.952	2.926	-1.12	NO_2…NO_2	A…A
5	x,y,z	O4	O6'	3.026	3.291	-0.47	NO_2…NO_2	A…A'
		O7	O6'	3.027	2.984	-1.17		
		O4	O7'	3.258	3.476	-0.33		
		O6	O7'	3.030	3.003	-1.16		
		O7	O7'	3.050	3.112	-0.96		
5^{p1-p2}	x,y,z	O7	O7"	2.415	–	–	Cannot exist	A…A'
5^{p2-p1}	x,y,z	O2A	O6'	2.865	–	–	NO_2…NO_2	A…A'
5^{p2-p2}	x,y,z	O2A	O7"	3.032	2.931	-1.41	NO_2…NO_2	A…A'
		O3A	O6"	3.441	2.977	-1.20		
		O4A	O6"	3.282	3.344	-0.42		
6	1+x,y,z	O6	O6'	3.399	3.560	-0.16	NO_2…NO_2	A…A'
		O6	O2'	3.644	3.670	-0.14		
		O6	O4'	4.233	3.940	-0.09		
6^{p1-p2}	1+x,y,z	O6	O2"	2.766	–	–	NO_2…NO_2	A…A'
6^{p2-p1}	1+x,y,z	No close Cont			–	–	vdW	A…A'
6^{p2-p2}	1+x,y,z	O4A	O2"	2.594	2.863	-1.36	NO_2…NO_2	A…A'
7	-1/2+x,1.5-y,-1/2+z	N6	N4'	3.120	3.121	-1.40	π…π stacking	A…A'
		N5	C3'	3.305				
		N4	N6'	3.145	3.138	-1.31		
		C3	N5'	3.296				

8	1/2+x,1.5-y,-1/2+z	N5<br>N6<br>N5<br>N4<br>O2<br>N5<br>N4<br>C1<br>C3	N3'<br>N5'<br>N6'<br>O3'<br>N4'<br>C3'<br>C1'<br>N4'<br>N5'	3.500<br>3.252<br>3.222<br>3.356<br>3.518<br>3.200<br>3.222<br>3.227<br>3.226	3.331<br>3.223<br>3.223<br>3.335<br>3.552	-0.91<br>-1.18<br>-1.23<br>-0.50<br>-0.27	π…π stacking	A…A'
9	-1+x,y,z	O1<br>O1<br>O5	H8B<br>H7A<br>H7B	2.92<br>2.56<br>2.64	2.90<br>2.56<br>2.68	-0.51<br>-1.36<br>-1.07	C-H…O	A…C
9^{p2-p2}	-1+x,y,z	O1<br>O1<br>O7A	H8B<br>H7A<br>H7B	2.92<br>2.56<br>3.64	2.90<br>2.56<br>2.46	-0.51<br>-1.36<br>-1.95	C-H…O	A…C
10	x,y,z	O1<br>O1<br>N1<br>N1<br>O3	H6B<br>H8C<br>H6B<br>H7C<br>H7B	2.67<br>2.58<br>2.62<br>3.25<br>3.39	2.48<br>2.64<br>2.67<br>3.24<br>3.28	-1.61<br>-1.00<br>-1.09<br>-0.29<br>-0.22	C-H…O(N)	A…C
10^{p2-p2}	x,y,z	O1<br>O1<br>N1<br>N1<br>O5A	H6B<br>H8C<br>H6B<br>H7C<br>H7C	2.67<br>2.58<br>2.62<br>3.25<br>3.14	2.48<br>2.64<br>2.67<br>3.24<br>2.64	-1.61<br>-1.00<br>-1.09<br>-0.29<br>-1.04	C-H…O(N)	A…C
11	1.5-x,1/2+y,1/2-z	N3<br>N4	H8A<br>H5A	2.51<br>3.93	2.59<br>2.99	-1.31<br>-0.52	C-H…N	A…C
12	1-x,1-y,1-z	O4	H6C	2.75	2.68	-1.01	C-H…O	A…C
12^{p2-p2}	1-x,1-y,1-z	O3A	H6C	2.69	2.72	-0.84	C-H…O	A…C
13	2-x,1-y,1-z	O4<br>O6<br>O3<br>O4<br>O3	H5B<br>H5B<br>H7B<br>H7B<br>H6C	2.69<br>3.36<br>2.53<br>2.95<br>2.87	2.72<br>3.24<br>2.44<br>2.78<br>2.97	-0.85<br>-0.21<br>-1.31<br>-0.76<br>-0.39	C-H…O	A…C
13^{p2-p2}	2-x,1-y,1-z	O3A<br>O4A<br>O4A<br>O6A	H5B<br>H5B<br>H6C<br>H7B	2.83<br>2.58<br>3.32<br>2.78	2.85<br>2.80<br>3.13<br>3.02	-0.53<br>-0.54<br>-0.25<br>-0.35	C-H…O	A…C
14	x,y,z	O1<br>N5	H5'A<br>H5'A	2.63<br>2.72	2.62<br>2.58	-1.34<br>-1.15	C-H…O(N)	A…C'
14^{p2-p2}	x,y,z	O1<br>O1	H7"A<br>H5'B	2.97<br>2.68	2.613<br>2.376	-1.21<br>-2.00	C-H…O	A…C'
15	1.5-x,1/2+y,1/2-z	O6<br>N2<br>O2	H8'C<br>H8'C<br>C8'	2.21<br>2.88<br>3.399	2.35<br>3.02<br>3.264	-2.00<br>-0.63<br>-0.78	C-H…O(N)	A…C'
15^{p2-p2}	1.5-x,1/2+y,1/2-z	N2<br>N3<br>O5A	H8"B<br>H5C<br>C8"	2.74<br>2.68<br>3.616	2.93<br>2.84<br>3.264	-0.92<br>-0.71<br>-0.72	C-H…O(N)	A…C'
16	1/2-x,1/2+y,1/2-z	N2<br>N2<br>N3<br>O7<br>O7	H8'A<br>H7'C<br>H7'B<br>H8'A<br>H6'B	2.88<br>2.69<br>2.87<br>2.52<br>2.89	2.88<br>2.99<br>2.77<br>2.49<br>2.88	-0.68<br>-0.56<br>-1.29<br>-1.18<br>-0.51	C-H…O(N)	A…C'
16^{p1-p2}	1/2-x,1/2+y,1/2-z	O7	H6"C	2.21	–	–	C-H…O	A…C'
16^{p2-p1}	1/2-x,1/2+y,1/2-z	O2A	H8'A	2.35	–	–	C-H…O	A…C'
16^{p2-p2}	1/2-x,1/2+y,1/2-z	O2A<br>O5A	H8"C<br>C8"	3.17<br>3.616	2.37<br>3.264	-1.63<br>-0.72	C-H…O	A…C'

Table S8. Pair intermolecular interaction energies (kcal/mol) and shortened contacts (Å) of anion A' (primed) with its closest environment for salt **1**.

Entry	Symmetry code	Atomic pair		Distance X-ray	Distance calc.	Energy	Type of interaction	Molecular pair
1'	-1+x,y,z	O2'	O5'	3.040	2.953	-1.20	NO_2…NO_2	A'…A'
		O3'	O5'	3.102	3.025	-1.06		
		O3'	O7'	3.114	3.171	-0.70		
1'$^{p1-p2}$	-1+x,y,z	O3'	O6"	3.358	–	–	vdW	A'…A'
1'$^{p2-p1}$	-1+x,y,z	O3"	O7'	2.835	–	–	NO_2…NO_2	A'…A'
1'$^{p2-p2}$	-1+x,y,z	O2"	O6"	3.415	3.041	-0.95	NO_2…NO_2	A'…A'
		O3"	O6"	3.092	3.291	-0.50		
2'	1+x,y,z	O5'	O2'	3.040	2.953	-1.20	NO_2…NO_2	A'…A'
		O5'	O3'	3.102	3.025	-1.06		
		O7'	O3'	3.114	3.171	-0.70		
2'$^{p1-p2}$	1+x,y,z	O7'	O3"	2.835	–	–	NO_2…NO_2	
2'$^{p2-p1}$	1+x,y,z	O6"	O3'	3.358	–	–	vdW	
2'$^{p2-p2}$	1+x,y,z	O6"	O2"	3.415	3.041	-0.95	NO_2…NO_2	
		O6"	O3"	3.092	3.291	-0.50		
3'	-x,2-y,1-z	O2'	O4'	2.957	3.270	-0.41	NO_2…NO_2	A'…A'
		O4'	O2'	2.957	3.270	-0.41		
		O2'	O2'	3.314	3.219	-0.47		
3'$^{p1-p2}$	-x,2-y,1-z	No close contact			–	–	vdW	A'…A'
3'$^{p2-p1}$	-x,2-y,1-z	No close contact			–	–	vdW	A'…A'
3'$^{p2-p2}$	-x,2-y,1-z	No close contact					vdW	A'…A'
4'	1-x,2-y,1-z	O5'	O5'	2.995	2.898	-1.77	NO_2…NO_2	A'…A'
		O5'	O4'	2.948	3.256	-0.31		
		O4'	O5'	2.948	3.256	-0.31		
4'$^{p1-p2}$	1-x,2-y,1-z	No close cont			–	–	vdW	A'…A'
4'$^{p2-p1}$	1-x,2-y,1-z	No close cont			–	–	vdW	A'…A'
4'$^{p2-p2}$	1-x,2-y,1-z	O4"	O4"	4.662	3.689	-0.13	NO_2…NO_2	A'…A'
		O4"	O7"	2.919	3.422	-0.26		
		O7"	O4"	2.919	3.422	-0.26		
5'	x,y,z	O6'	O4	3.026	3.291	-0.47	NO_2…NO_2	A'…A
		O6'	O7	3.027	2.984	-1.17		
		O7'	O4	3.258	3.476	-0.33		
		O7'	O6	3.030	3.003	-1.16		
		O7'	O7	3.050	3.112	-0.96		
5'$^{p1-p2}$	x,y,z	O6'	O2A	2.865	–	–	NO_2…NO_2	A'…A
5'$^{p2-p1}$	x,y,z	O7"	O7	2.415	–	–	Cannot exist	A'…A
5'$^{p2-p2}$	x,y,z	O7"	O2A	3.032	2.931	-1.41	NO_2…NO_2	A'…A
		O6"	O3A	3.441	2.977	-1.20		
		O6"	O4A	3.282	3.344	-0.42		
6'	-1+x,y,z	O6'	O6	3.399	3.560	-0.16	NO_2…NO_2	A'…A
		O2'	O6	3.644	3.670	-0.14		
		O4'	O6	4.233	3.940	-0.09		
6'$^{p1-p2}$	-1+x,y,z	No close cont			–	–	vdW	
6'$^{p2-p1}$	-1+x,y,z	O2"	O6	2.766	–	–	NO_2…NO_2	A'…A
6'$^{p2-p2}$	-1+x,y,z	O2"	O4A	2.594	2.863	-1.36	NO_2…NO_2	A'…A
7'	1/2+x,1.5-y,1/2+z	N6'	N4	3.145	3.138	-1.31	π…π stacking	A'…A
	1/2+x,1.5-y,1/2+z	N5'	C3	3.296				
	1/2+x,1.5-y,1/2+z	N4'	N6	3.120	3.121	-1.40		
	1/2+x,1.5-y,1/2+z	C3'	N5	3.305				

8'	-1/2+x,1.5-y,1/2+z	N3'	N5	3.500	3.331	-0.91	π…π stacking	A'…A
		N5'	N6	3.252	3.223	-1.18		
		N6'	N5	3.222	3.223	-1.23		
		O3'	N4	3.356	3.335	-0.50		
		N4'	O2	3.518	3.552	-0.27		
		N5'	C3	3.226				
		N4'	C1	3.227				
		C1'	N4	3.222				
		C3'	N5	3.200				
9'	1-x,1-y,1-z	N1'	H6A	2.57	2.53	-1.36	C-H…O(N)	A'…C
		O3'	H5A	2.75	2.71	-0.76		
		O6'	C5	3.698	3.536	-0.44		
		N1'	H5A	3.13	3.10	-0.41		
		O1'	H8A	2.69	2.61	-1.15		
		O6'	C6	3.486	3.326	-0.82		
9'$^{p2-p2}$	1-x,1-y,1-z	O3"	H5A	2.43	3.00	-0.45	C-H…O	A'…C
		O2"	C5	3.33	3.43	-0.63		
10'	2-x,1-y,1-z	O1'	H5C	2.72	2.95	-0.62	C-H…O(N)	A'…C
		N1'	H5C	2.72	2.86	-0.75		
		O7'	H5B	2.71	2.76	-0.95		
11'	-1/2+x,1.5-y,1/2+z	N3'	H8C	2.58	2.51	-1.60	C-H…N	A'…C
		N4'	H7C	2.84	2.71	-1.00		
12'	1-x,1-y,1-z	O1'	H7'B	2.40	2.33	-1.82	C-H…O(N)	A'…C'
		N5'	H8'B	2.48	2.49	-1.56		
		O1'	H5'C	2.98	2.67	-0.82		
		O1'	H8'B	2.90	2.78	-0.79		
12'$^{p2-p2}$	1-x,1-y,1-z	O1'	H7"B	2.73	2.16	-2.90	C-H…O(N)	A'…C'
		N5'	H8"A	3.41	2.54	-1.34		
13'	-1/2+x,1.5-y,1/2+z	O4'	H6'C	2.46	2.49	-1.25	C-H…O(N)	A'…C'
		O3'	H6'A	2.61	2.71	-0.84		
		N3'	H5'A	3.12	3.05	-0.67		
		O4'	H5'B	3.09	2.93	-0.47		
		N2'	H5'B	2.82	2.81	-0.95		
13'$^{p1-p2}$	-1/2+x,1.5-y,1/2+z	No close cont			–	–	vdW	A'…C'
13'$^{p2-p1}$	-1/2+x,1.5-y,1/2+z	O4"	H6'C	2.41	–	–	C-H…O	A'…C'
13'$^{p2-p2}$	-1/2+x,1.5-y,1/2+z	O3"	H6"A	4.15	2.775	-0.65	C-H…O(N)	A'…C'
		O4"	H6"B	2.74	2.389	-1.70		
		N2'	H5'A	2.85	3.121	-0.49		
		N3'	H5'B	3.19	2.980	-0.63		
14'	1/2+x,1.5-y,1/2+z	O5'	H6'A	2.66			C-H…O(N)	A'…C'
		O5'	C6'	3.082	3.177	-1.01		
		N3'	H7'A	2.58	2.41	-1.85		
		N2'	H6'A	3.00	2.90	-0.65		
		O4'	H6'B	3.57	3.02	-0.47		
14'$^{p2-p2}$	1/2+x,1.5-y,1/2+z	N3'	H7"A	2.50	2.48	-1.58	C-H…O(N)	A'…C'
		N2'	H6"A	3.70	2.94	-0.61		
		O5"	H6"C	3.17	2.66	-1.42		
15'	1/2-x, 1/2+y, 1/2-z	O2'	H6'C	3.11	3.18	-0.21	C-H…O	A'…C'
15'$^{p2-p2}$	1/2-x, 1/2+y, 1/2-z	O7"	H6"C	2.93	2.65	-0.92	C-H…O	A'…C'

Table S9. Pair intermolecular interaction energies (kcal/mol) and shortened contacts (Å) of cation C (unprimed) with its closest environment for salt **1**.

Entry	Symmetry code	Atomic pair		Distance X-ray	Distance calc.	Energy	Type of interaction	Molecular pair
1	1+x,y,z	H8B	O1	2.92	2.90	-0.51	C-H…O	C…A
		H7A	O1	2.56	2.56	-1.36		
		H7B	O5	2.64	2.68	-1.07		
1^{p2-p2}	1+x,y,z	H8B	O1	2.92	2.90	-0.51	C-H…O	C…A
		H7A	O1	2.56	2.56	-1.36		
		H7B	O7A	3.64	2.46	-1.95		
2	x,y,z	H6B	O1	2.67	2.48	-1.61	C-H…O(N)	C…A
		H8C	O1	2.58	2.64	-1.00		
		H6B	N1	2.62	2.67	-1.09		
		H7C	N1	3.25	3.24	-0.29		
		H7B	O3	3.39	3.28	-0.22		
2^{p2-p2}	x,y,z	H6B	O1	2.67	2.48	-1.61	C-H…O(N)	C…A
		H8C	O1	2.58	2.64	-1.00		
		H6B	N1	2.62	2.67	-1.09		
		H7C	N1	3.25	3.24	-0.29		
		H7C	O5A	3.14	2.64	-1.04		
3	1.5-x,-1/2+y,1/2-z	H8A	N3	2.51	2.59	-1.31	C-H…N	C…A
		H5A	N4	3.93	2.99	-0.52		
4	1-x,1-y,1-z	H6C	O4	2.75	2.68	-1.01	C-H…O	C…A
4^{p1-p2}	1-x,1-y,1-z	H6C	O3A	2.69	2.72	-0.84	C-H…O	C…A
5	2-x,1-y,1-z	H5B	O4	2.69	2.72	-0.85	C-H…O	C…A
		H5B	O6	3.36	3.24	-0.21		
		H7B	O3	2.53	2.44	-1.31		
		H7B	O4	2.95	2.78	-0.76		
		H6C	O3	2.87	2.97	-0.39		
5^{p2-p2}	2-x,1-y,1-z	H5B	O3A	2.83	2.85	-0.53	C-H…O	C…A
		H5B	O4A	2.58	2.80	-0.54		
		H6C	O4A	3.32	3.13	-0.25		
		H7B	O6A	2.78	3.02	-0.35		
6	1-x,1-y,1-z	H6A	N1'	2.57	2.53	-1.36	C-H…O(N)	C…A'
		H5A	O3'	2.75	2.71	-0.76		
		C5	O6'	3.698	3.536	-0.44		
		H5A	N1'	3.13	3.10	-0.41		
		H8A	O1'	2.69	2.61	-1.15		
		C6	O6'	3.486	3.326	-0.82		
6^{p1-p2}	1-x,1-y,1-z	H5A	O3''	2.43	3.00	-0.45	C-H…O	C…A'
		C5	O2''	3.33	3.43	-0.63		
7	2-x,1-y,1-z	H5C	O1'	2.72	2.95	-0.62	C-H…O(N)	C…A'
		H5C	N1'	2.72	2.86	-0.75		
		H5B	O7'	2.71	2.76	-0.95		
8	1/2+x,1.5-y,-1/2+z	H8C	N3'	2.58	2.51	-1.60	C-H…N	C…A'
		H7C	N4'	2.84	2.71	-1.00		
9	1+x,y,z	H8B	H7'B	2.38	2.24	-1.19	vdW	C…C'
10	x,y,z	H8C	H7'B	2.93	2.71	-0.42	vdW	C…C'

Table S10. Pair intermolecular interaction energies (kcal/mol) and shortened contacts (Å) of cation C' (primed) with its closest environment for salt **1**.

Entry	Symmetry code	Atomic pair		Distance X-ray	Distance calc.	Energy	Type of interaction	Molecular pair
1'	x,y,z	H5'A	O1	2.63	2.62	-1.34	C-H…O(N)	C'…A
		H5'A	N5	2.72	2.58	-1.15		
$1'^{p2-p2}$	x,y,z	H7"A	O1	2.97	2.613	-1.21	C-H…O	C'…A
		H5"B	O1	2.68	2.376	-2.00		
2'	1.5-x,-1/2+y,1/2-z	H8'C	O6	2.21	2.35	-2.00	C-H…O(N)	C'…A
		H8'C	N2	2.88	3.02	-0.63		
		C8'	O2	3.399	3.264	-0.78		
$2'^{p2-p2}$	1.5-x,-1/2+y,1/2-z	H8"B	N2	2.74	2.93	-0.92	C-H…O(N)	C'…A
		H5"C	N3	2.68	2.84	-0.71		
		C8"	O5A	3.616	3.264	-0.72		
3'	1/2-x,-1/2+y,1/2-z	H8'A	N2	2.88	2.88	-0.68	C-H…O(N)	C'…A
		H7'C	N2	2.69	2.99	-0.56		
		H7'B	N3	2.87	2.77	-1.29		
		H8'A	O7	2.52	2.49	-1.18		
		H6'B	O7	2.89	2.88	-0.51		
$3'^{p1-p2}$	1/2-x,-1/2+y,1/2-z	H8'A	O2A	2.35	–	–	C-H…O	C'…A
$3'^{p2-p1}$	1/2-x,-1/2+y,1/2-z	H6"C	O7	2.21	–	–	C-H…O	C'…A
$3'^{p2-p2}$	1/2-x,-1/2+y,1/2-z	H8"C	O2A	3.17	2.37	-1.63	C-H…O	C'…A
		C8"	O5A	3.616	3.264	-0.72		
4'	1-x,1-y,1-z	H7'B	O1'	2.40	2.33	-1.82	C-H…O(N)	C'…A'
		H8'B	N5'	2.48	2.49	-1.56		
		H5'C	O1'	2.98	2.67	-0.82		
		H8'B	O1'	2.90	2.78	-0.79		
$4'^{p2-p2}$	1-x,1-y,1-z	H7"B	O1'	2.73	2.16	-2.90	C-H…O(N)	C'…A'
		H8"A	N5'	3.41	2.54	-1.34		
5'	-1/2+x,1.5-y,-1/2+z	H6'C	O4'	2.46	2.49	-1.25	C-H…O(N)	C'…A'
		H6'A	O3'	2.61	2.71	-0.84		
		H5'A	N3'	3.12	3.05	-0.67		
		H5'B	O4'	3.09	2.93	-0.47		
		H5'B	N2'	2.82	2.81	-0.95		
$5'^{p2-p1}$	-1/2+x,1.5-y,-1/2+z	No close cont			–	–	vdW	C'…A'
$5'^{p1-p2}$	-1/2+x,1.5-y,-1/2+z	H6'C	O4"	2.41	–	–	C-H…O	C'…A'
$5'^{p2-p2}$	-1/2+x,1.5-y,-1/2+z	H6"A	O3"	4.15	2.775	-0.65	C-H…O(N)	C'…A'
		H6"B	O4"	2.74	2.389	-1.70		
		H5"A	N2'	2.85	3.121	-0.49		
		H5"B	N3'	3.19	2.980	-0.63		
6'	1/2+x,1.5-y,-1/2+z	H6'A	O5'	2.66			C-H…O(N)	C'…A'
		C6'	O5'	3.082	3.177	-1.01		
		H7'A	N3'	2.58	2.41	-1.85		
		H6'A	N2'	3.00	2.90	-0.65		
		H6'B	O4'	3.57	3.02	-0.47		
$6'^{p2-p2}$	1/2+x,1.5-y,-1/2+z	H7"A	N3'	2.50	2.48	-1.58	C-H…O(N)	C'…A'
		H6"A	N2'	3.70	2.94	-0.61		
		H6"C	O5"	3.17	2.66	-1.42		
7'	1/2-x, 1/2+y, 1/2-z	H6'C	O2'	3.11	3.18	-0.21	C-H…O	C'…A'
$7'^{p2-p2}$	1/2-x, 1/2+y, 1/2-z	H6"C	O7"	2.93	2.65	-0.92	C-H…O	C'…A'
8'	-1+x,y,z	H7'B	H8B	2.38	2.24	-1.19	vdW	C'…C
9'	x,y,z	H7'B	H8C	2.93	2.71	-0.42	vdW	C'…C

Table S11. Pair intermolecular interaction energies (kcal/mol) and shortened contacts (Å) of molecule of BTF with its closest environment in the crystal of BTF **2**.

Entry	Symmetry code	Atomic pair		Distance X-ray	Distance calc.	Energy[a]	Energy[b]	Type of interaction
1	-1+x,y,-1+z	O4	O5	3.063	3.049	-0.76	-0.1	van-der-Waals
		O4	O6	3.178	3.240	-0.54		
2	1+x,y,1+z	O5	O4	3.063	3.049	-0.76	-0.1	van-der-Waals
		O6	O4	3.178	3.240	-0.54		
3	-1+x,y,z	O1	O5	3.131	3.169	-0.74	-3.7	π…π stacking
		N2	O6	3.186	3.215	-0.88		
		N2	N5	3.235				
4	1+x,y,z	O5	O1	3.131	3.169	-0.74	-3.7	π…π stacking
		O6	N2	3.186	3.215	-0.88		
		N5	N2	3.235				
5	x,y,-1+z	O3	O5	2.992			-5.7	π…π stacking
		O3	N6	2.949	2.938	-2.13		
		O4	N1	3.044				
		O4	C1	3.050	3.033	-1.54		
		O4	C6	3.160				
		N3	N6	3.043				
		O3	N6	3.814	3.892	-0.11		
6	x,y,1+z	O5	O3	2.992			-5.7	π…π stacking
		N1	O4	3.044				
		N6	O3	2.949	2.938	-2.13		
		N6	N3	3.043				
		C1	O4	3.050	3.033	-1.54		
		C6	O4	3.160				
		N6	O3	3.814	3.892	-0.11		
7	-x,-y,-1/2+z	O1	O2	2.972	3.112	-0.78	-0.2	van-der-Waals
		N2	O1	3.141	3.333	-0.44		
		N2	O2	3.288	3.245	-0.76		
8	-x,-y,1/2+z	O2	O1	2.972	3.112	-0.78	-0.2	van-der-Waals
		O1	N2	3.141	3.333	-0.44		
		O2	N2	3.288	3.245	-0.76		
9	1-x,-y,-1/2+z	O1	N6	2.946	2.924	-1.91	-5.6	O(N)…π
		N2	N6	3.161				
		C1	O2	3.080	2.964	-2.06		
		C5	O2	3.083				
		C6	O2	2.942				
10	1-x,-y,1/2+z	O2	C1	3.080	2.964	-2.06	-5.6	O(N)…π
		O2	C5	3.083				
		O2	C6	2.942				
		N6	O1	2.946	2.924	-1.91		
		N6	N2	3.161				
11	-1/2+x,1/2-y,z	O3	N4	3.125			-3.7	O(N)…π
		O4	O3	2.907				
		O4	N4	3.002				
		N3	N4	2.853	2.865	-2.45		
		C4	O6	3.074	2.991	-1.48		
12	1/2+x,1/2-y,z	O3	O4	2.907			-3.7	O(N)…π
		O6	C4	3.074	2.991	-1.48		
		N4	O3	3.125				
		N4	O4	3.002				
		N4	N3	2.853	2.865	-2.45		
13	-1/2+x,1/2-y,-1+z	O3	O6	3.345	3.322	-0.40	-0.5	van-der-Waals
14	1/2+x,1/2-y,1+z	O6	O3	3.345	3.322	-0.40	-0.5	van-der-Waals

[a] obtained from the EML correlation; [b] obtained from the $E_{int} = E_{AB} - E_A - E_B$ formula

Table S12. Pair intermolecular interaction energies (kcal/mol) and shortened contacts (Å) of anion A (unprimed) with its closest environment for the co-crystal **3**.

Entry	Symmetry code	Atomic pair		Distance X-ray	Distance calc.	Energy	Type of interaction	Molecular pair
1	-1+x,y,z	N6	O2	3.077	3.096	1.14	weak O…π	A…A
		O5	O3	3.276	3.173	0.58		
		N1	O3	3.818	3.897	0.14		
2	1+x,y,z	O2	N6	3.077	3.096	1.14	weak O…π	A…A
		O3	O5	3.276	3.173	0.58		
		O3	N1	3.818	3.897	0.14		
3	2-x,-y,1-z	O6	O6	2.972	2.955	1.40	NO_2…NO_2	A…A
		O6	O3	3.177	3.233	0.59		
		O3	O6	3.177	3.233	0.59		
4	2-x,-y,1-z	O7	O7	3.448	3.269	0.44	NO_2…NO_2	A…A
		O7	N1	3.453	3.390	0.45		
		N1	O7	3.453	3.390	0.45		
5	1+x,-1+y,z	O5	N2'	3.362	3.403	0.50	NO_2…NO_2<br>weak O…π	A…A'
		O5	O7'	3.398	3.460	0.26		
		O4	O4'	3.662	3.595	0.20		
		O4	C3'	3.296	3.213	0.73		
		N2	O4'	3.243	3.242	0.66		
		N2	O7'	3.482	3.501	0.33		
6	2+x,-1+y,z	O4	O6'	2.875	2.942	1.24	NO_2…NO_2	A…A'
		O2	O7'	3.322	3.299	0.51		
		O3	O7'	3.305	3.281	0.52		
7	x,y,z	N4	H5A	2.41	2.30	2.59	C-H…N	A…C
		N4	H7A	2.59	2.57	1.39		
		N3	H6A	2.77	2.30	0.53		
8	1+x,y,z	N2	H7C	2.45	2.47	1.69	C-H…O(N)	A…C
		N3	H7C	2.73	2.73	1.02		
		N3	H8B	2.84	2.60	1.18		
		O2	H7A	2.74	2.83	0.77		
9	x,-1+y,z	O5	H7'B	2.65	2.61	1.16	C-H…O	A…C'
		O5	H5'C	3.16	2.84	0.60		
		O7	H5'C	3.10	3.04	0.33		
10	1+x,-1+y,z	O7	H6'B	2.79	2.78	0.72	C-H…O	A…C'
		O3	H6'B	3.12	2.79	0.67		
		O3	H7'B	2.48	2.51	1.12		
		O4	H6'B	3.32	3.13	0.40		
		O4	H7'B	2.94	2.95	0.54		
11	-x,1-y,1-z	O1	H6'C	2.78	2.67	0.82	C-H…O	A…C'
12	1-x,1-y,1-z	O6	H6'B	2.47	2.45	1.65	C-H…O(N)	A…C'
		O6	H5'C	2.51	2.55	1.36		
		N1	H5'B	2.91	2.80	1.09		
		O1	H5'B	2.77	2.83	1.03		
13	x,y,z	O1	O6"	3.040			O(N)…π	A…M
		O1	N5"	2.939	2.926	1.87		
		O1	C4"	3.253				
		O1	C5"	3.033				
		N5	C1"	3.176				
		N5	C2"	3.048				
		N5	C3"	3.014	3.014	2.03		
		N5	C4"	3.138				
		N5	C5"	3.297				
		N4	O1"	3.868	4.204	0.05		
14	1+x,y,z	C1	O3"	3.134	3.118	0.77	weak O…π	A…M
		N4	O4"	3.084				
		N5	O4"	3.118	3.121	1.33		

Table S13. Pair intermolecular interaction energies (kcal/mol) and shortened contacts (Å) of anion A' (primed) with its closest environment in the co-crystal **3**.

Entry	Symmetry code	Atomic pair		Distance X-ray	Distance calc.	Energy	Type of interaction	Molecular pair
1	-1+x,y,z	O2'	O1'	2.988	3.054	0.98	weak O…π	A'…A'
		O6'	N6'	3.158	3.142	0.85		
2	1+x,y,z	O1'	O2'	2.988	3.054	0.98	weak O…π	A'…A'
		N6'	O6'	3.158	3.142	0.85		
3	-1-x,2-y,-z	O5'	O5'	2.957	2.925	1.23	NO_2…NO_2	A'…A'
		O3'	O5'	3.300	3.254	0.49		
		O5'	O3'	3.300	3.254	0.49		
4	-1+x,1+y,z	N2'	O5	3.362	3.403	0.50	NO_2…NO_2<br>weak O…π	A'…A
		O7'	O5	3.398	3.460	0.26		
		O4'	O4	3.662	3.595	0.20		
		C3'	O4	3.296	3.213	0.73		
		O4'	N2	3.243	3.242	0.66		
		O7'	N2	3.482	3.501	0.33		
5	2+x,-1+y,z	O6'	O4	2.875	2.942	1.24	NO_2…NO_2	A'…A
		O7'	O2	3.322	3.299	0.51		
		O7'	O3	3.305	3.281	0.52		
6	-1+x,1+y,z	O5'	H6C	2.71	2.94	0.49	C-H…O	A'…C
		O3'	H6C	3.03	3.01	0.35		
		O3'	H7B	2.89	3.04	0.34		
		O7'	C7	3.37	3.41	0.63		
7	x,1+y,z	O5'	H8C	2.77	2.67	1.01	C-H…O	A'…C
		O4'	H7B	2.66	2.56	1.39		
8	-1-x,1-y,-z	O2'	H8A	2.69	2.68	1.15	C-H…O	A'…C
		O3'	H8C	2.70	2.71	1.06		
9	-x,1-y,-z	N1'	H6B	2.69	2.60	1.23	C-H…O(N)	A'…C
		O2'	H6B	2.48	2.59	1.45		
		O1'	H8A	2.56	2.62	0.94		
		O5'	H6C	2.88	2.94	0.44		
10	-1+x,y,z	N2'	H7'A	2.49	2.53	1.80	C-H…O(N)	A'…C'
		N3'	H7'A	2.42	2.34	2.15		
		O6'	H7'C	2.76	2.81	0.83		
11	x,y,z	N4'	H7'C	2.56	2.42	1.90	C-H…N	A'…C'
		N4'	H8'B	2.55	2.54	1.41		
		N3'	H6'A	2.80	2.78	0.83		
12	-x,1-y,1-z	N4'	H8'C	2.56	2.67	1.29	C-H…N	A'…C'
13	-1+x,y,z	N6'	O5''	3.227			O(N)…π	A'…M
		C3'	O5''	3.015	3.055	1.01		
		N6'	N6''	3.216	3.182	1.21		
		C1'	N6''	3.244				
		O2'	O2''	3.184	3.054	0.47		
14	x,y,z	O1'	C1''	3.108			O(N)…π	A'…M
		O1'	C2''	3.038	2.954	1.73		
		O1'	C3''	3.194				
		O1'	C6''	3.334				
		N5'	N5''	3.021				
		N5'	C4''	3.145	2.983	2.09		
		N5'	C5''	3.001				

Table S14. Pair intermolecular interaction energies (kcal/mol) and shortened contacts (Å) of cation C (unprimed) with its closest environment in the co-crystal **3**.

Entry	Symmetry code	Atomic pair		Distance X-ray	Distance calc.	Energy	Molecular pair	Type of interaction
1	x,y,z	H5A	N4	2.41	2.30	2.59	C…A	C-H…N
		H7A	N4	2.59	2.57	1.39		
		H6A	N3	2.77	2.30	0.53		
2	-1+x,y,z	H7C	N2	2.45	2.47	1.69	C…A	C-H…O(N)
		H7C	N3	2.73	2.73	1.02		
		H8B	N3	2.84	2.60	1.18		
		H7A	O2	2.74	2.83	0.77		
3	1+x,-1+y,z	H6C	O5'	2.71	2.94	0.49	C…A'	C-H…O
		H6C	O3'	3.03	3.01	0.35		
		H7B	O3'	2.89	3.04	0.34		
		C7	O7'	3.37	3.41	0.63		
4	x,-1+y,z	H8C	O5'	2.77	2.67	1.01	C…A'	C-H…O
		H7B	O4'	2.66	2.56	1.39		
5	-1-x,1-y,-z	H8A	O2'	2.69	2.68	1.15	C…A'	C-H…O
		H8C	O3'	2.70	2.71	1.06		
6	-x,1-y,-z	H6B	N1'	2.69	2.60	1.23	C…A'	C-H…O(N)
		H6B	O2'	2.48	2.59	1.45		
		H8A	O1'	2.56	2.62	0.94		
		H6C	O5'	2.88	2.94	0.44		
7	x,y,z	H5A	N2"	2.78	2.83	0.96	C…M	C-H…O(N)
		H5B	O4"	2.61	2.46	1.45		
8	-x,1-y,-z	H5C	N2"	2.66	2.54	1.48	C…M	C-H…O(N)
		H8A	O1"	3.51	3.23	0.29		
9	1-x,1-y,-z	H5C	O2"	2.57	2.50	1.56	C…M	C-H…O
		H6B	O2"	2.56	2.52	1.58		

Table S15. Pair intermolecular interaction energies (kcal/mol) and shortened contacts (Å) of cation C' (primed) with its closest environment in the co-crystal **3**.

Entry	Symmetry code	Atomic pair		Distance X-ray	Distance calc.	Energy	Molecular pair	Type of interaction
1'	x,1+y,z	H7'B	O5	2.649	2.615	1.16	C'…A	C-H…O
		H5'C	O5	3.162	2.839	0.60		
		H5'C	O7	3.103	3.036	0.33		
2'	-1+x,1+y,z	H6'B	O7	2.797	2.783	0.72	C'…A	C-H…O
		H6'B	O3	3.119	2.794	0.67		
		H7'B	O3	2.485	2.512	1.12		
		H6'B	O4	3.317	3.136	0.40		
		H7'B	O4	2.944	2.952	0.54		
3'	-x,1-y,1-z	H6'C	O1	2.780	2.672	0.82	C'…A	C-H…O
4'	1-x,1-y,1-z	H6'B	O6	2.470	2.449	1.65	C'…A	C-H…O(N)
		H5'C	O6	2.512	2.547	1.36		
		H5'B	N1	2.911	2.794	1.09		
		H5'B	O1	2.771	2.835	1.03		
5	1+x,y,z	H7'A	N2'	2.486	2.533	1.80	C'…A'	C-H…O(N)
		H7'A	N3'	2.418	2.341	2.15		
		H7'C	O6'	2.760	2.812	0.83		
6'	x,y,z	H7'C	N4'	2.560	2.421	1.90	C'…A'	C-H…N
		H8'B	N4'	2.551	2.541	1.41		
		H6'A	N3'	2.806	2.757	0.83		
7'	-x,1-y,1-z	H8'C	N4'	2.559	2.670	1.29	C'…A'	C-H…N
8'	x,y,z	H8'B	O6"	2.94	2.97	0.50	C'…M	C-H…O
9'	-x,1-y,1-z	H6'C	N4"	2.66	2.68	0.96	C'…M	C-H…O(N)
		H6'C	O6"	2.76	2.80	0.82		
		H5'B	N4"	2.75	2.68	0.99		
		H8'C	N4"	2.93	2.61	1.13		
		H8'C	O6"	3.00	2.93	0.62		
10'	1-x,1-y,1-z	H5'A	O6"	3.05	3.04	0.36	C'…M	C-H…O
		H8'C	O5"	3.42	3.39	0.14		

Table S16. Pair intermolecular interaction energies (kcal/mol) and shortened contacts (Å) of BTF molecule (M) with its closest environment in the co-crystal **3**.

Entry	Symmetry code	Atomic pair		Distance X-ray	Distance calc.	Energy	Molecular pair	Type of interaction
1	-1+x,y,z	O3"	N6"	3.020	3.039	1.46	M…M	weak π…π Stacking
		O4"	O2"	3.155	3.122	1.21		
2	1+x,y,z	N6"	O3"	3.020	3.039	1.46	M…M	weak π…π Stacking
		O2"	O4"	3.155	3.122	1.21		
3	1-x,1-y,-z	O1"	O1"	3.191	3.467	0.46	M…M	van-der-Waals
4	x,y,z	O6"	O1	3.040			M…A	O(N)…π
		N5"	O1	2.939	2.926	1.87		
		C4"	O1	3.253				
		C5"	O1	3.033				
		C1"	N5	3.176				
		C2"	N5	3.048				
		C3"	N5	3.014	3.014	2.03		
		C4"	N5	3.138				
		C5"	N5	3.297				
		O1"	N4	3.868	4.204	0.05		
5	-1+x,y,z	O4"	N4	3.084			M…A	weak O…π
		O4"	N5	3.118	3.121	1.33		
		O3"	C1	3.134	3.118	0.77		
6	1+x,y,z	O5"	N6'	3.227			M…A'	O(N)…π
		O5"	C3'	3.015	3.055	1.01		
		N6"	N6'	3.216	3.182	1.21		
		N6"	C1'	3.244				
		O2"	O2'	3.184	3.054	0.47		
7	x,y,z	C1"	O1'	3.108			M…A'	O(N)…π
		C2"	O1'	3.038	2.954	1.73		
		C3"	O1'	3.194				
		C6"	O1'	3.335				
		N5"	N5'	3.021				
		C4"	N5'	3.145	2.983	2.09		
		C5"	N5'	3.001				
8	x,y,z	N2"	H5A	2.78	2.83	0.96	M…C	C-H…O(N)
		O4"	H5B	2.61	2.46	1.45		
9	-x,1-y,-z	N2"	H5C	2.66	2.54	1.48	M…C	C-H…O(N)
		O1"	H8A	3.51	3.23	0.29		
10	1-x,1-y,-z	O2"	H5C	2.57	2.50	1.56	M…C	C-H…O
		O2"	H6B	2.56	2.52	1.58		
11	x,y,z	O6"	H8'B	2.94	2.97	0.50	M…C'	C-H…O
12	-x,1-y,1-z	N4"	H6'C	2.66	2.68	0.96	M…C'	C-H…O(N)
		O6"	H6'C	2.76	2.80	0.82		
		N4"	H5'B	2.75	2.68	0.99		
		N4"	H8'C	2.93	2.61	1.13		
		O6"	H8'C	3.00	2.93	0.62		
13	1-x,1-y,1-z	O6"	H5'A	3.05	3.04	0.36	M…C'	C-H…O
		O5"	H8'C	3.42	3.39	0.14		

References

1. Frisch, M. J.; Trucks, G. W.; Schlegel, H. B.; Scuseria, G. E.; Robb, M. A.; Cheeseman, J. R.; Montgomery, J. A.; Kudin, K. N., Jr.; Burant, J. C.; Millam, J. M.; Iyengar, S. S.; Tomasi, J.; Barone, V.; Mennucci, B.; Cossi, M.; Scalmani, G.; Rega, N.; Petersson, G. A.; Nakatsuji, H.; Hada, M.; Ehara, M.; Toyota, K.; Fukuda, R.; Hasegawa, J.; Ishida, M.; Nakajima, T.; Honda, Y.; Kitao, O.; Nakai, H.; Klene, M.; Li, X.; Knox, J. E.; Hratchian, H. P.; Cross, J. B.; Bakken, V.; Adamo, C.; Jaramillo, J.; Gomperts, R.; Stratmann, R. E.; Yazyev, O.; Austin, A. J.; Cammi, R.; Pomelli, C.; Ochterski, J. W.; Ayala, P. Y.; Morokuma, K.; Voth, G. A.; Salvador, P.; Dannenberg, J. J.; Zakrzewski, V. G.; Dapprich, S.; Daniels, A. D.; Strain, M. C.; Farkas, O.; Malick, D. K.; Rabuck, A. D.; Raghavachari, K.; Foresman, J. B.; Ortiz, J. V.; Cui, Q.; Baboul, A. G.; Clifford, S.; Cioslowski, J.; Stefanov, B. B.; Liu, G.; Liashenko, A.; Piskorz, P.; Komaromi, I.; Martin, R. L.; Fox, D. J.; Keith, T.; Al-Laham, M. A.; Peng, C. Y.; Nanayakkara, A.; Challacombe, M.; Gill, P. M. W.; Johnson, B.; Chen, W.; Wong, M. W.; Gonzalez, C.; Pople, J. A. *Gaussian 03, Revision E.01*, Gaussian, Inc.: Wallingford, 2004.
2. Bader, R. F. W. *Atoms in Molecules. A Quantum Theory*, Clarendon Press, Oxford, 1990.
3. Keith, T. A. **2014**, *AIMAll*, *Version 14.11.23*. TK Gristmill Software, Overland Park KS, USA (http://aim.tkgristmill.com)
4. T. Lu, F. Chen, *J. Comput. Chem.* **2012**, *33*, 580-592.
